# Diversity and Abundance of Drosophilidae (Diptera) in Post-Fire Forest Ecosystems of Central European Russia

**DOI:** 10.3390/insects17090892

**Published:** 2026-08-25

**Authors:** Nikolai G. Gornostaev, Alexander B. Ruchin, Mikhail N. Esin, Oleg E. Lazebny, Alex M. Kulikov, Anatoliy A. Khapugin

**Affiliations:** 1 N.K. Koltzov Institute of Developmental Biology, Russian Academy of Sciences (RAS), 119334 Moscow, Russia; n_gornostaev@mail.ru (N.G.G.); o.e.lazebny@idbras.ru (O.E.L.); amkulikov@gmail.com (A.M.K.); 2 Joint Directorate of the Mordovia State Nature Reserve and National Park “Smolny”, 430005 Saransk, Russia; esinmishka@gmail.com (M.N.E.); hapugin88@yandex.ru (A.A.K.); 3 Institute of Environmental and Agricultural Biology (X-BIO), Tyumen State University, 625003 Tyumen, Russia

**Keywords:** Drosophilidae, post-fire fauna, habitat associations, seasonal dynamics, Republic of Mordovia

## Abstract

Fire can produce long-lasting changes in forest habitat structure and insect communities, but responses may depend on local post-fire conditions rather than fire history alone. We tested two working hypotheses: (1) drosophilid assemblages at fire-affected sites would differ in abundance and species composition from those in unburned forest, and (2) selected species would show consistent associations with fire-affected or unburned habitats and could therefore serve as candidate indicators of post-fire conditions. Drosophilids were sampled at 11 forest sites in the Mordovia State Nature Reserve, Central European Russia: three sites burned in 2010, six burned in both 2010 and 2021, and two unburned controls. From May to September 2022, 3537 individuals representing 30 species and nine genera were collected using beer-baited traps. The two control sites yielded 2175 individuals (61% of the total catch). Restricted permutation analyses that retained sampling site as the spatial unit and accounted for repeated monthly observations confirmed significant differences in community composition between fire-affected and unburned sites; the contrast remained significant when relative abundances were analyzed, showing that it was not explained solely by differences in total catch. In contrast, once- and twice-burned sites did not differ significantly when compared directly. Seven drosophilid species, *Drosophila histrio*, *D. testacea*, *D. phalerata*, *D. transversa*, *D. obscura*, *D. subobscura*, and *Leucophenga quinquemaculata*, showed their strongest site-level associations with unburned forest and were 8- to 74-fold more abundant there than at fire-affected sites. However, none of these species-level associations remained significant after false-discovery-rate correction. *Gitona distigma* was nearly four-fold more abundant at fire-affected sites but likewise was not statistically validated as an indicator. These results support a persistent association between fire-related habitat transformation and drosophilid assemblage structure, while indicating that the number of fires alone did not account for heterogeneity among post-fire sites. We conclude that drosophilid assemblages respond sensitively to fire history and habitat structure, and that the species showing strong habitat associations may be useful candidates for biomonitoring post-fire forest recovery in Central European Russia, provided that the results are confirmed by more long-term and comprehensive studies.

## Simple Summary

When a forest burns, the insects living there lose their food sources, shelter, and breeding sites. Recovery can take many years, but exactly how long and which species come back first is still poorly understood for many insect groups. We looked at small flies called drosophilids in a nature reserve in Central European Russia where wildfires struck in 2010 and again in 2021. Using simple baited traps hung in the trees from May to September 2022, we caught about 3500 flies belonging to 30 species. The unburned parts of the forest had far more flies and a different mix of species than the burned areas. These patterns held even 12 years after the first fire, suggesting that some drosophilid species may serve as useful candidate indicators of how well a forest is recovering after burning. We caution, however, that our study covered only one year and used a single trap type at each site, so the results should be confirmed by longer, more comprehensive surveys before they are applied in management.

## 1. Introduction

Fire has shaped terrestrial ecosystems for millions of years. Contemporary fire ecology has moved away from the earlier view of fire as a purely destructive and irreversible disturbance [1] toward recognition of fire as an integral ecological process that influences most terrestrial ecosystems worldwide [2,3]. Hundreds of thousands of fires occur globally each year [2,4], yet their effects on ecosystems, biodiversity, carbon stocks, and climate are frequently underestimated [5]. Fire is a major driver of population dynamics and species diversity in both plants and animals [6,7,8,9] and can also contribute to soil degradation [10]. Biodiversity responses to fire depend on fire-regime attributes such as frequency, intensity, and spatial extent [9,11,12]. These attributes also shape post-fire vegetation recovery and, consequently, the recovery of associated animal communities. Comparative studies of plant and animal communities exposed to different fire frequencies and histories are therefore particularly important [13,14].

Arthropods are integral components of forest ecosystems and interact closely with a wide range of plants and animals [15,16,17,18]. Consequently, major disturbances such as fire can alter the abundance and diversity of numerous arthropod groups [19,20]. For example, a study conducted in a hemiboreal old-growth forest in Tyresta, Sweden, documented high species diversity and abundance of phorid flies (Diptera: Phoridae) following fire [21]. In fire-affected pine forests, phorid assemblages contained similar numbers of species across burned sites but were less diverse than assemblages in unburned forests. Nevertheless, several species reached their highest abundances in burned habitats. Approximately three years after fire, phorid assemblages had become broadly similar to those found in unburned forests [22].

Studies conducted in the eastern North American pine barrens have documented both positive and negative effects of fire on predatory and parasitoid Diptera [23], with fire-adapted species generally being less severely affected. Smoke may also act as an attractant for Diptera associated with burned habitats [24]. Following an accidental fire, the species diversity of tephritid flies (Diptera: Tephritidae) was higher after the fire than during the pre-fire period. The authors attributed this increase to greater habitat heterogeneity created by the disturbance [25]. Similarly, a study in Brazil showed that fire induced profuse flowering in certain plant species, which positively affected the abundance of tephritids associated with their inflorescences [26]. By contrast, total Diptera abundance declined following fire in deciduous forests in Zambia [27]. In Central Europe, Diptera abundance varied substantially among fire-affected sites [28]; across forest types, catches were generally highest during the first year after fire.

The present study aimed to assess the abundance and species diversity of drosophilid flies (Diptera: Drosophilidae) collected using beer-baited traps in forests of the Mordovia State Nature Reserve affected by fires in 2010 and 2021. Specifically, we aimed to (1) characterize drosophilid species diversity at fire-affected and unburned sites, (2) compare community composition and abundance between these site categories while accounting for repeated seasonal sampling, and (3) identify species associated with particular post-fire or unburned habitat conditions. We tested two working hypotheses: first, that fire-affected sites would support lower drosophilid catches and assemblages differing in species composition from those of unburned forest; and second, that some drosophilid species would show consistent associations with fire-affected or unburned habitats and could therefore serve as candidate indicators of post-fire forest conditions. Because the study included sites burned once and twice, we also evaluated whether these two post-fire categories differed in assemblage composition, without assuming a priori that repeated fire would necessarily produce a stronger response.

## 2. Materials and Methods

### 2.1. Study Area and Sampling Sites

The study was conducted in 2022 in the Mordovia State Nature Reserve, European Russia. The reserve lies in the transition zone between the southern taiga and forest-steppe. The mean annual air temperature is approximately 4.7 °C, and annual precipitation ranges from 406.6 to 681.3 mm [29]. Most of its territory is covered by forest ecosystems, predominantly pine forests. Following the wildfires of 2010, many pine stands were destroyed or substantially altered. In areas where pine stands were lost, young forests dominated by *Betula pendula* subsequently developed [29].

**Figure 1:**
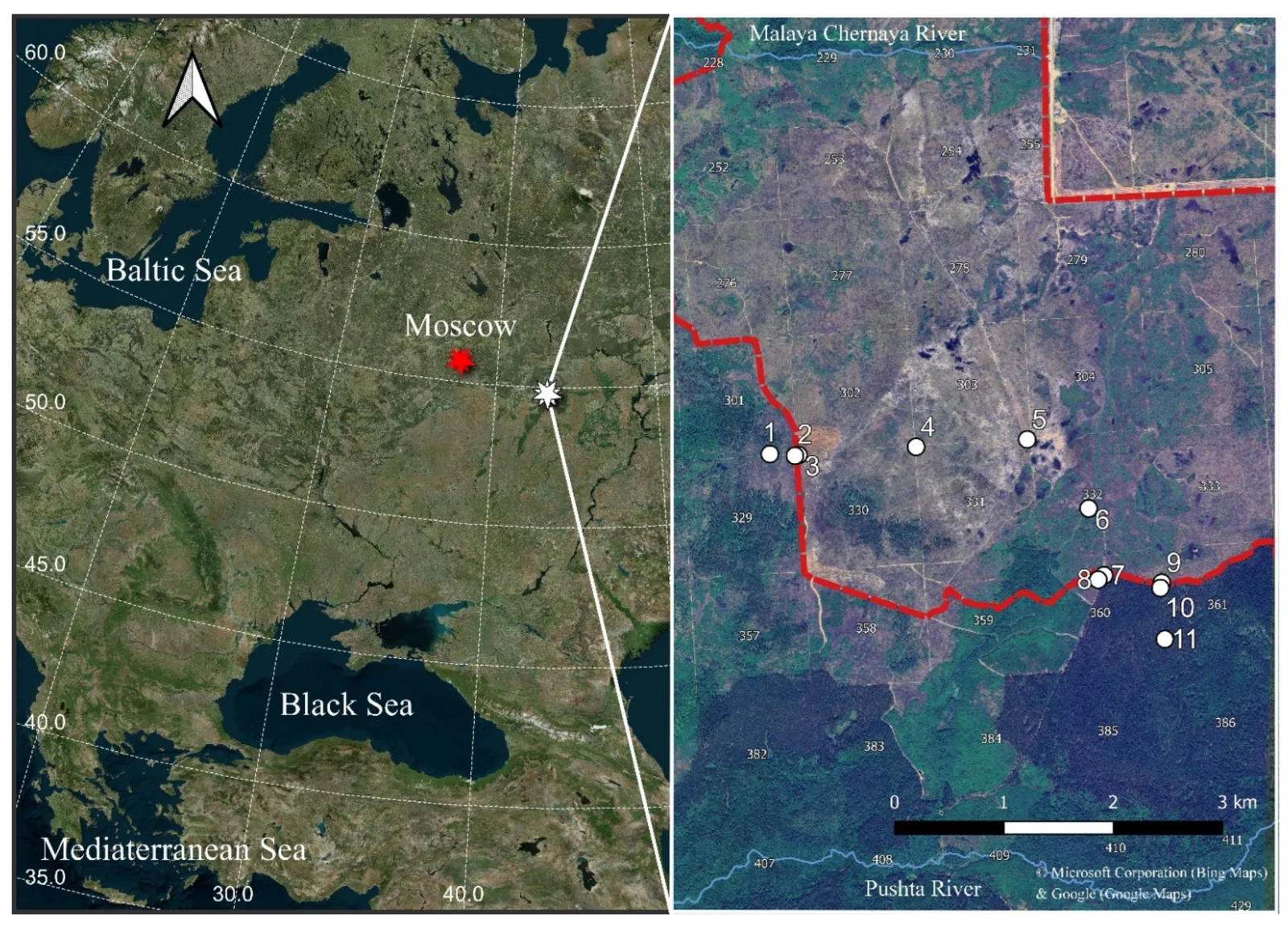
Geographic location of the Mordovia State Nature Reserve in Europe. Study sites are indicated by white dots and labeled according to the site numbering used in the text. The red line denotes the boundary of the 2021 fire.

A total of 11 sampling sites were selected (Figure 1). The sites differed in fire history, burn severity, distance from the fire perimeter, and the extent of vegetation recovery following the 2010 fires. Unburned forest sites were included as controls [30]. Prior to the fires, the study area formed a continuous mixed-forest tract, and the locations subsequently sampled had a highly similar floristic composition. The two unburned control sites were selected specifically to represent this pre-fire habitat type and to match the fire-affected forest as closely as possible in vegetation composition. A summary of fire history, position relative to the fire perimeter, and major habitat characteristics of all sampling sites is provided in Table 1.

Vegetation cover–abundance data recorded using the Braun–Blanquet scale are provided for all sampling sites in Table S1.

### 2.2. Sampling

Sampling was conducted from May to September 2022 using beer-baited fermentation traps. One trap was installed at each sampling site at a height of 1.5 m on a small wooden tripod. Each trap consisted of a 5 L plastic container with an opening cut into one side approximately 10 cm above the bottom [31] and contained approximately 500 mL of light beer mixed with 50 g of sugar. The bait was replaced at each of the 13 sampling visits, which were conducted at approximately two-week intervals during the study period.

Drosophilid specimens were identified by N.G. Gornostaev using the identification key provided in [32]. All specimens of Drosophilidae collected during this study are preserved in 70% ethanol and deposited in the entomological collection of the N.K. Koltzov Institute of Developmental Biology, Russian Academy of Sciences (IDB RAS), Moscow, Russia, and are available for examination upon request. The classification and nomenclature of Drosophilidae followed Grimaldi [33].

### 2.3. Statistical Analysis

For seasonal site-level species abundances, we calculated the mean, median, standard deviation, quartiles, skewness, and kurtosis (Table S2).

Similarity in species composition among sampling sites was evaluated using hierarchical agglomerative clustering in Statistica 12 (StatSoft Inc., Tulsa, OK, USA) [34]. Pairwise dissimilarities were calculated using Euclidean distances, and clusters were formed using the complete-linkage method.

Temporal and spatial patterns in species occurrence and abundance were visualized using diagrams prepared in Microsoft Excel 2019. To illustrate differences associated with fire history, samples collected during the same month were grouped according to the fire histories of the sampling sites. To illustrate seasonal patterns, data from sites with similar fire histories were compared among sampling months. Species represented by fewer than five individuals across all samples were excluded from these visualizations.

Community-level differences in drosophilid assemblages were evaluated using restricted permutational multivariate analysis based on Bray–Curtis dissimilarities [35,36]. For each sampling site and month, the abundances of the 30 recorded species were treated as a multivariate community vector. Species counts were square-root transformed before analysis to reduce the influence of highly abundant species while retaining information on both abundance and species composition. The pseudo-F statistic was calculated from the Gower-centered matrix of squared Bray–Curtis dissimilarities by comparing reduced and full distance-based models.

Differences associated with fire history were evaluated while accounting for seasonal variation by including sampling month as a nuisance factor. Sampling site was treated as the spatial observational unit. During permutations, fire-history labels were reassigned only among whole sampling sites, so that all repeated monthly observations from the same site retained the same permuted group label. Because the number of sites was small, exact permutation distributions were used for the principal between-site comparisons: burned versus unburned sites (55 possible allocations), the three fire-history categories—unburned, burned once, and burned twice (4620 allocations)—and once- versus twice-burned sites within the fire-affected area (84 allocations).

Seasonal variation in community composition was evaluated using a repeated-measures permutation design. Sampling site was included as a blocking factor, and month labels were permuted only within individual sites (4999 restricted permutations). Effect sizes are reported as partial R^2^, calculated as SS_effect/(SS_effect + SS_residual). To determine whether differences among fire-history categories were attributable only to variation in total trap catches, all community-level analyses were repeated using relative species abundances, thereby retaining differences in species composition while removing differences in total abundance. Seasonal variation in total drosophilid abundance and species richness was additionally evaluated using Friedman repeated-measures tests. Complete permutation and sensitivity-analysis results are provided in Table S4.

Community diversity was characterized using the Shannon diversity index and Simpson dominance index. The relative contribution of individual species to each community was evaluated using the Paliy–Kownacki dominance index [37,38].

Associations between drosophilid abundance and fire-history groups were evaluated using the group-equalized Indicator Value statistic (IndVal.g) following the framework implemented in the R package indicspecies [39,40]. To avoid temporal pseudoreplication, monthly drosophilid counts were summed within each sampling site before this analysis, leaving the 11 sampling sites as the independent spatial units. For the three-group classification (unburned, burned once, and burned twice), associations with individual groups only were considered (equivalent to max.order = 1 in multipatt). Because group sizes were small, statistical significance was evaluated using an exact permutation distribution containing all 4620 allocations that preserved the observed group sizes (2, 3, and 6 sites, respectively). A complementary binary analysis of burned versus unburned sites was evaluated using all 55 possible allocations of two control labels among the 11 sites. Raw *p* values were adjusted across the 30 drosophilid species using the Benjamini–Hochberg procedure. The vegetation indicator analysis, based on one vegetation record per sampling site, retained the original three-group classification and 9999 permutations.

Categorical principal component analysis was used to visualize relationships between species abundances and habitat categories. The proportion of variation in species abundances associated with differences among sampling sites was estimated using bootstrap resampling with 1000 replicates.

## 3. Results

### 3.1. Faunal Composition and Distribution of Drosophilids Across Post-Fire and Control Sites

A total of 3537 drosophilid individuals representing 30 species from nine genera were collected across the 11 forest sites, including both fire-affected and unburned control sites. Of the drosophilids captured in beer-baited traps, eight species from four genera belonged to the subfamily Steganinae, whereas 22 species from five genera belonged to the subfamily *Drosophilinae* (Table 2).

Seven species were represented by more than 100 individuals: *Drosophila testacea* (911 individuals), *Drosophila obscura* (821), *Gitona distigma* (454), *Drosophila transversa* (399), *Drosophila subobscura* (260), *Amiota albilabris* (126), and *Amiota rufescens* (123). Five species had intermediate abundances of 20–100 individuals: *Drosophila phalerata* (91 individuals), *Leucophenga quinquemaculata* (78), *Drosophila histrio* (70), *Scaptodrosophila rufifrons* (51), and *Chymomyza costata* (38). The remaining 18 species were each represented by fewer than 20 individuals and were therefore considered rare in the collected material (Table 2).

Detailed data on drosophilid captures at each site and in each sampling month from May to September are provided in Table S3.

### 3.2. Abundance and Species Diversity of Drosophilidae

**Figure 2:**
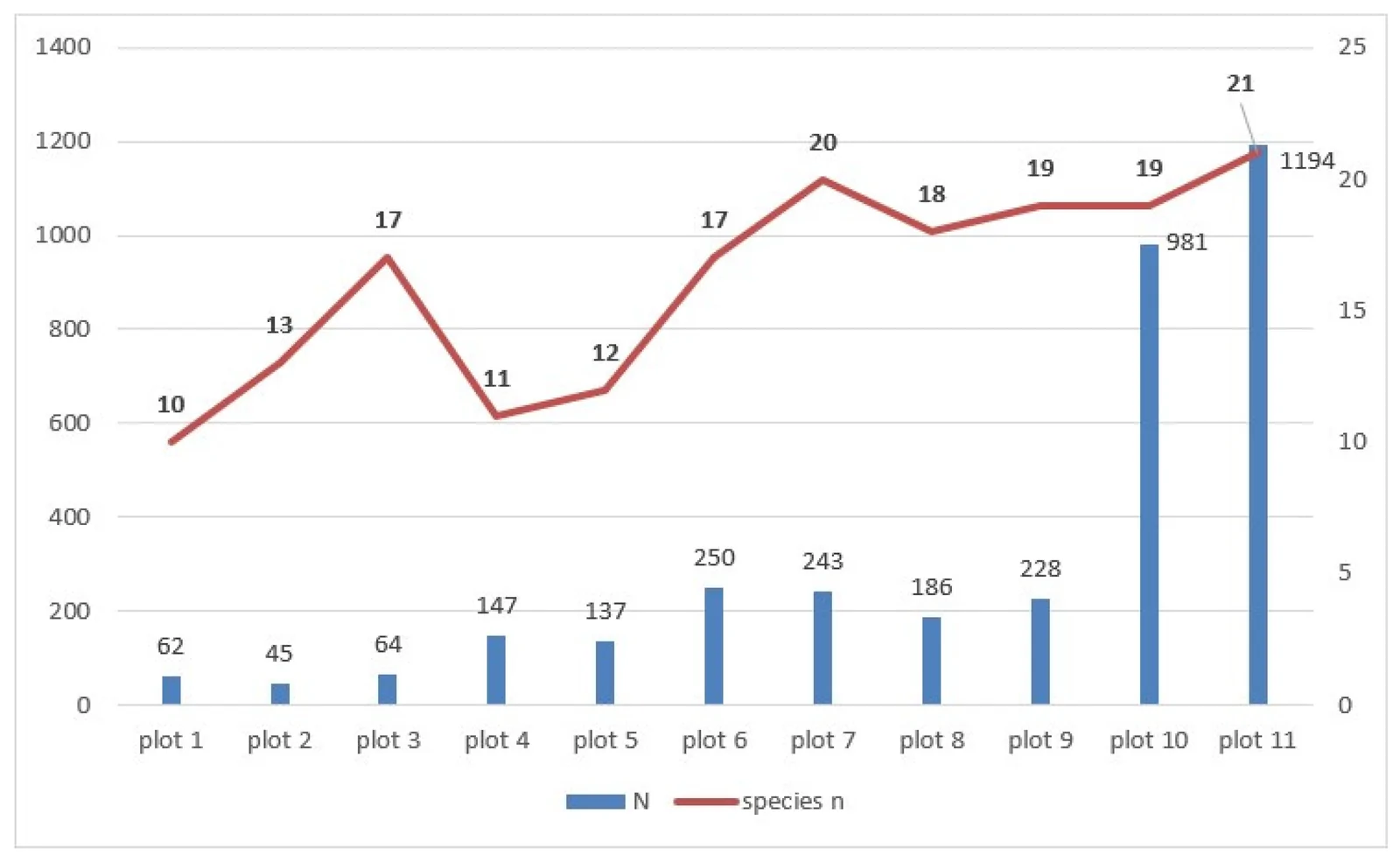
Total abundance of drosophilids (Diptera: Drosophilidae) collected using beer-baited fermentation traps across 11 study sites over the sampling season (May–September 2022) in the Mordovia State Nature Reserve, Republic of Mordovia, Central European Russia. Sites 1–9 are post-fire habitats; Sites 10 and 11 are unburned controls. The number of individuals recorded at each site is indicated above the corresponding bars. The number of drosophilid species recorded at each site is indicated above the red line on the graph.

A total of 3537 drosophilid individuals were collected across all 11 sites during the sampling season. Of these, 2175 individuals, representing 61% of the total catch, were captured at the two unburned control sites. Seasonal totals at control Sites 10 and 11 were 981 and 1194 individuals, respectively, substantially exceeding the abundance recorded at any of the post-fire sites (Figure 2; Table 2).

In descending order of abundance, the control sites were followed by Sites 6–9, at which 250, 243, 186, and 228 individuals were collected, respectively. With the exception of Site 8, these sites had burned twice but were only partially affected by the second fire. Sites 7–9 were also located close to the perimeter of the 2021 fire. Site 6 was the only exception: it was located 1500 m inside the fire perimeter but occupied a wet, low-lying habitat and experienced a low-severity fire in 2021. The lowest drosophilid abundances were recorded at Sites 1 and 2, which burned only in 2010, with 62 and 45 individuals, respectively, and at Sites 3–5, which burned in both 2010 and 2021, with 64, 147, and 137 individuals, respectively.

In terms of species richness, Sites 7–9 were broadly comparable to the control sites: 20, 18, and 19 species were recorded at Sites 7, 8, and 9, respectively, compared with 19 and 21 species at control Sites 10 and 11. The lowest species diversity was recorded at Sites 1 and 2, with 10 and 13 species, respectively. These sites were characterized by dense regeneration of young birch. Completely burned Sites 4 and 5 supported 11 and 12 species, respectively; these values were within the lower range observed among the fire-affected sites rather than uniquely low. Both sites were located well inside the area affected by the repeated fire of 2021 (Figure 2; Table 2).

### 3.3. Comparison of Drosophilid Species Composition Between Post-Fire and Control Sites

**Figure 3:**
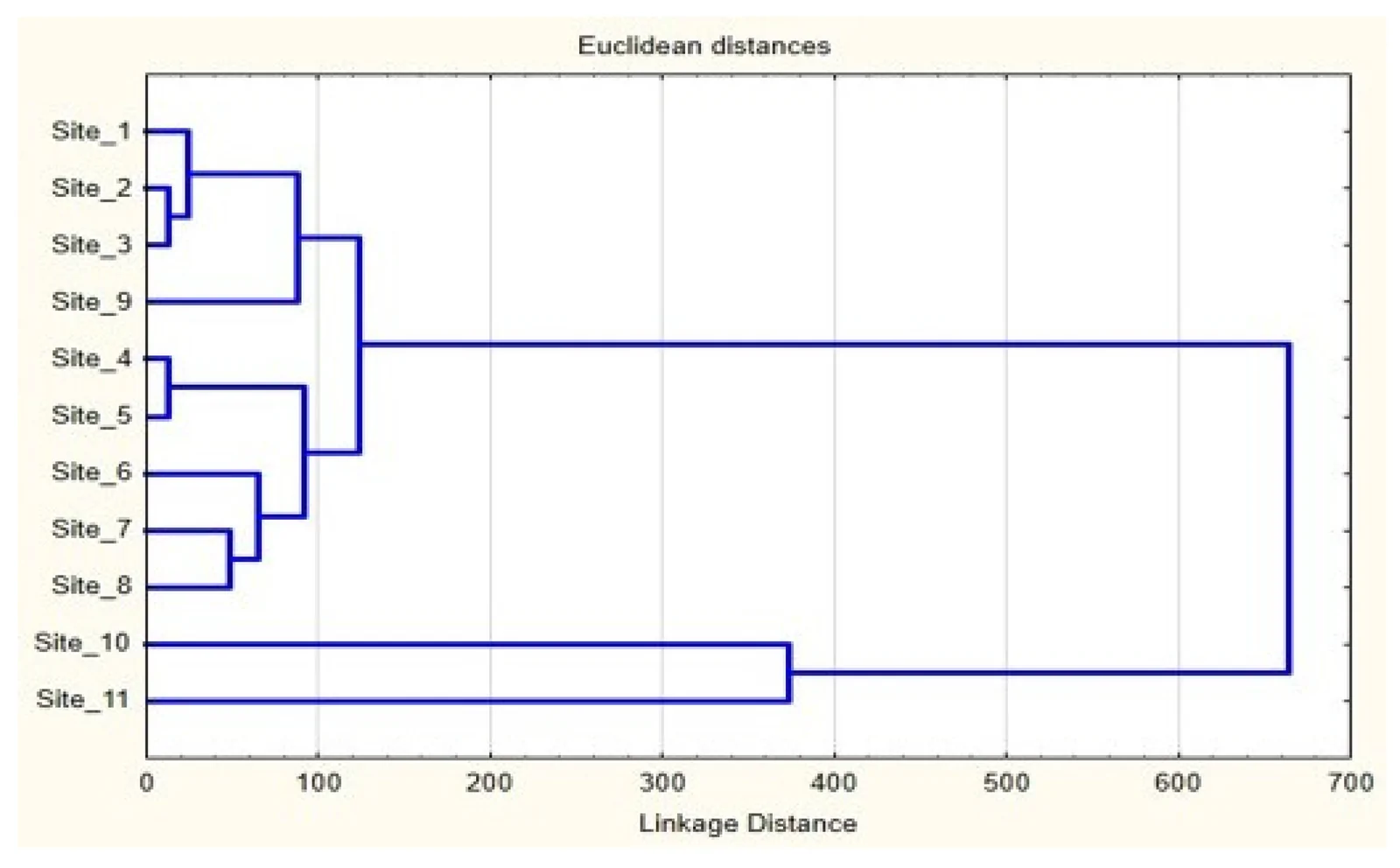
Hierarchical clustering of 11 study sites in the Mordovia State Nature Reserve, Republic of Mordovia, Central European Russia, based on drosophilid species abundances in beer-baited trap catches collected May–September 2022. Sites 1–9 are post-fire habitats; Sites 10 and 11 are unburned controls. Pairwise Euclidean distances were clustered using the complete-linkage method; the cophenetic correlation coefficient was r = 0.941.

Hierarchical cluster analysis based on species composition separated the sampling sites into two main groups: post-fire Sites 1–9 and unburned control Sites 10 and 11 (Figure 3). Within the post-fire group, differences in the time elapsed since the most recent fire did not consistently determine similarity in species composition. Sites 1 and 2, which burned only in 2010, clustered with Sites 3 and 9, which burned in both 2010 and 2021. Similarly, Site 8, which was affected only by the 2010 fire, clustered with Sites 6 and 7, which burned again in 2021.

Overall, the species composition of the unburned control sites differed more strongly from that of the post-fire sites than the post-fire sites differed from one another. The cophenetic correlation coefficient for the Euclidean-distance/complete-linkage dendrogram was 0.941, indicating close correspondence between the dendrogram and the original pairwise distance structure.

This separation was supported by the restricted permutation analysis that accounted for repeated monthly observations. Assemblages at the nine fire-affected sites differed significantly from those at the two unburned control sites after adjustment for sampling month (pseudo-F_1,49_ = 11.597, partial R^2^ = 0.191, exact *p* = 0.0182). The difference remained significant when species counts were converted to relative abundances (pseudo-F_1,49_ = 10.225, partial R^2^ = 0.173, exact *p* = 0.0182), showing that the contrast involved species composition and was not attributable solely to the much larger total catches at the control sites.

The overall comparison among unburned, once-burned, and twice-burned sites was also significant (square-root-transformed abundances: pseudo-F_2,48_ = 6.896, partial R^2^ = 0.223, exact *p* = 0.0130; relative abundances: pseudo-F_2,48_ = 5.899, partial R^2^ = 0.197, exact *p* = 0.0286). However, when the two post-fire categories were compared directly after excluding the unburned controls, assemblages at once- and twice-burned sites did not differ significantly (square-root-transformed abundances: pseudo-F_1,39_ = 1.818, partial R^2^ = 0.045, exact *p* = 0.333; relative abundances: pseudo-F_1,39_ = 1.408, partial R^2^ = 0.035, exact *p* = 0.512). Thus, the strongest community-level contrast was between unburned forest and the fire-affected area, whereas heterogeneity within the post-fire landscape was not explained simply by the number of fires experienced by a site.

**Figure 4:**
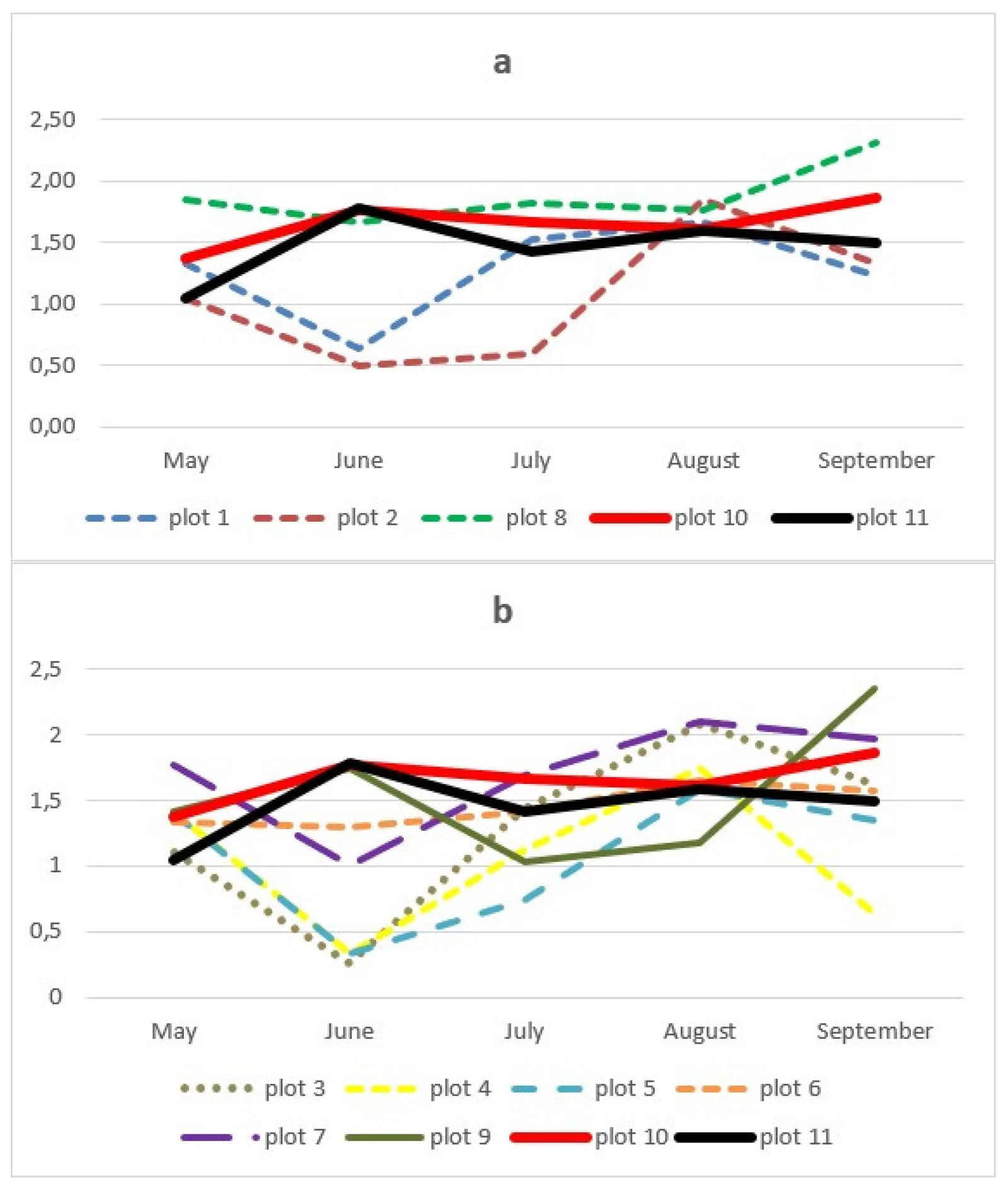
Seasonal changes in the Shannon diversity index. (**a**) Sites burned in 2010 and control sites; (**b**) sites burned twice (2010 and 2021) and control sites. Trends for control sites are shown by thick red and black lines.

Seasonal variation in drosophilid diversity at post-fire and unburned control sites was evaluated using the Shannon diversity index (Figure 4).

Sites affected only by the 2010 fire exhibited two contrasting seasonal patterns (Figure 4a). At Sites 1 and 2, diversity declined sharply in early summer from values initially comparable to those of the control sites. It subsequently increased markedly during the second half of summer before declining again in September (Table S3). These fluctuations may reflect temporal variation in the availability of fermenting substrates and fungi, which constitute important resources for many drosophilid species. Such resources may be less abundant and more variable in fire-affected communities characterized by reduced food availability and altered vegetation.

By contrast, diversity at Site 8 remained relatively stable throughout the sampling period. Shannon index values at this site were generally comparable to those recorded at the control sites and were slightly higher during spring and autumn. This pattern may indicate a more advanced stage of forest recovery at Site 8 (Table S3).

Sites affected by repeated fires in 2010 and 2021 also showed considerable variation in the seasonal trajectories of drosophilid diversity (Figure 4b). Sites 3–5, which were characterized by relatively low drosophilid abundances, exhibited the most pronounced seasonal fluctuations in diversity, broadly resembling those observed at Sites 1 and 2. Site 7 showed a similar seasonal pattern but supported a greater total abundance and higher species richness. During the second half of summer and in autumn, diversity at Site 7 exceeded that recorded at the unburned control sites.

Seasonal changes in Shannon diversity at Sites 6 and 9 were generally similar to those observed at control Sites 10 and 11. The principal differences were a more pronounced decline in early summer at Site 6 and a stronger decline during the second half of summer at Site 9 (Figure 4b; Table S3).

### 3.4. Dominance Structure of Drosophilid Assemblages at Post-Fire and Control Sites

**Figure 5:**
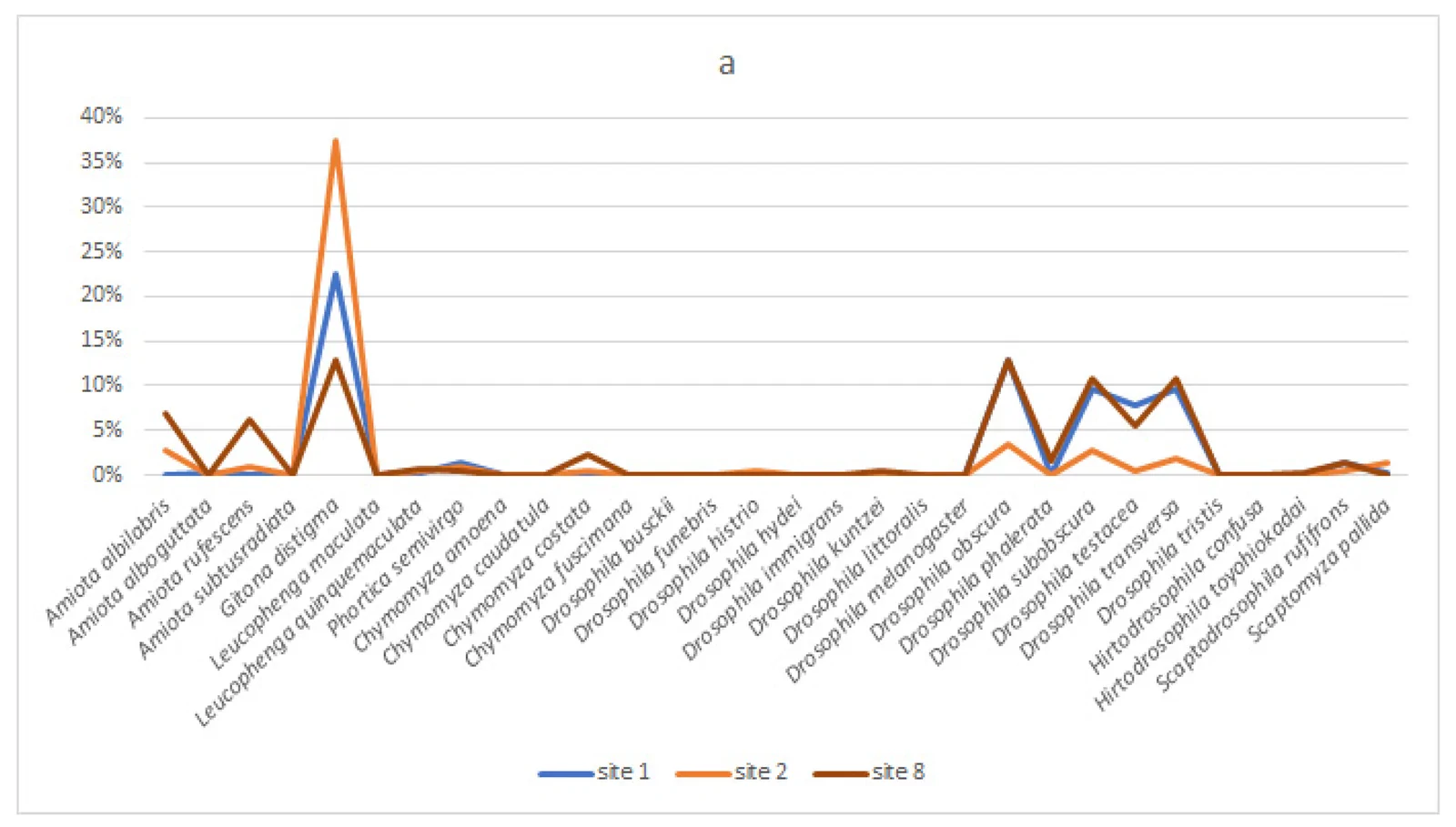

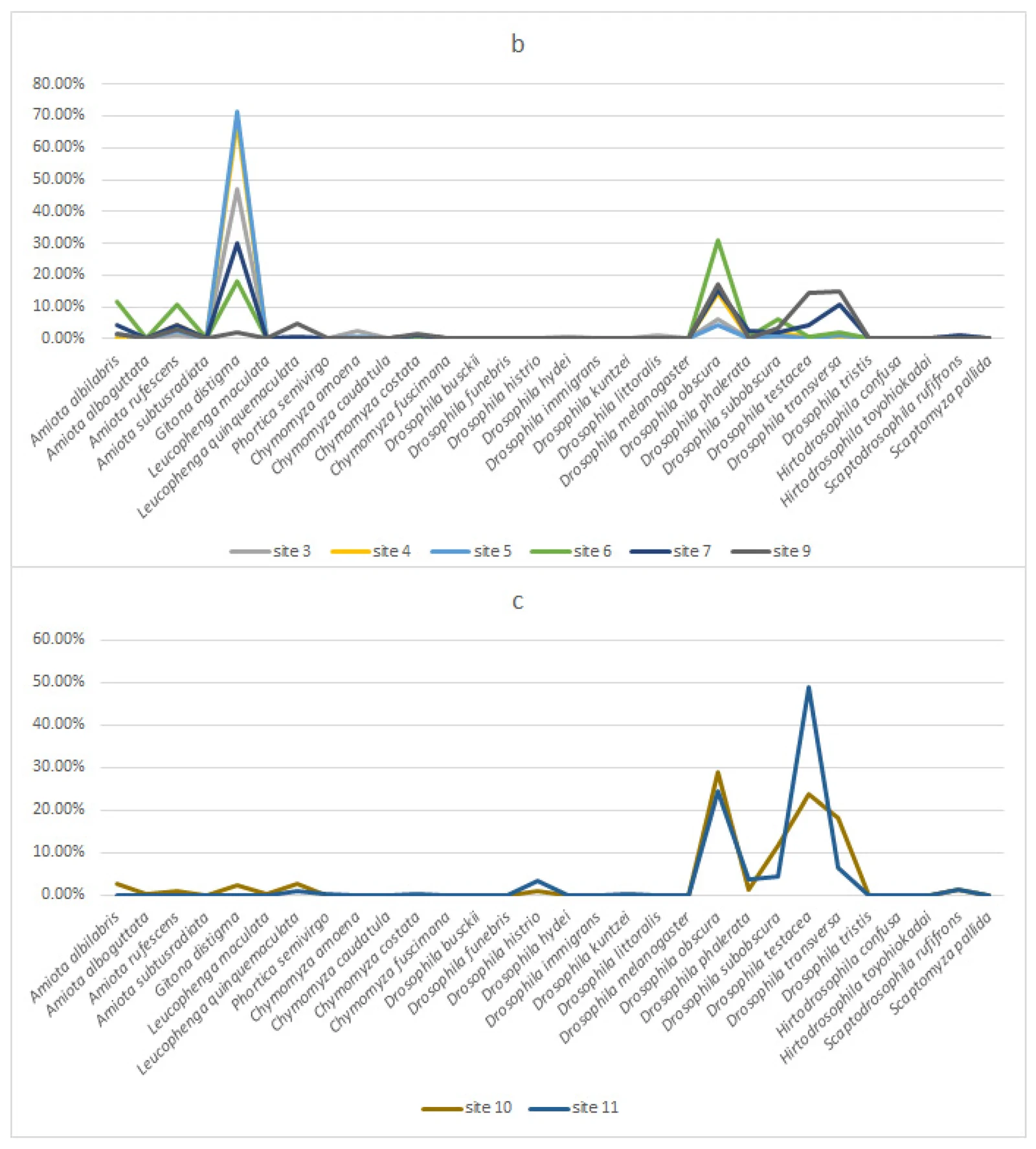
Species dominance structure of drosophilid communities (Diptera: Drosophilidae) based on total abundance per site, estimated using the Paliy–Kownacki dominance index from beer-baited trap catches collected May–September 2022 in the Mordovia State Nature Reserve, Republic of Mordovia, Central European Russia: (**a**) sites burned in 2010; (**b**) sites burned twice (2010 and 2021); (**c**) unburned control sites.

The contributions of individual species to drosophilid community structure were assessed using the Paliy–Kownacki dominance index (Di) (Figure 5). The main difference between post-fire and control forest sites was associated with *Gitona distigma*, which reached substantially higher abundances at post-fire sites, particularly at those that burned twice. By contrast, unburned control sites were characterized by very high abundances of the dominant species *Drosophila testacea*, *D. obscura*, and *D. transversa*, whose numbers exceeded those at post-fire sites by several fold.

**Figure 6:**
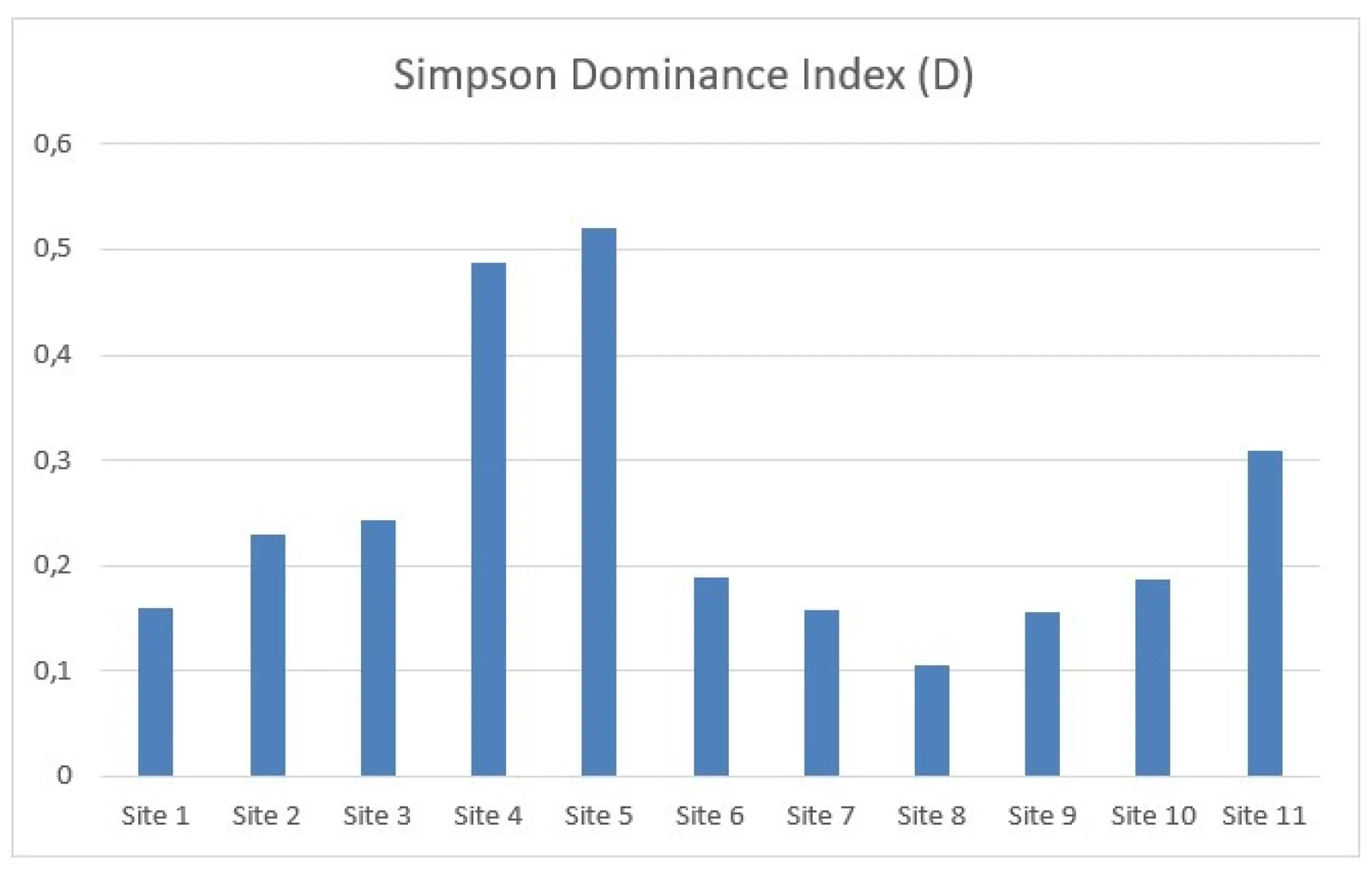
Simpson’s dominance index based on total drosophilid abundance (Diptera: Drosophilidae) at each of 11 study sites in the Mordovia State Nature Reserve, Republic of Mordovia, Central European Russia, calculated from beer-baited trap catches collected May–September 2022. Sites 1–9 are post-fire habitats; Sites 10 and 11 are unburned controls.

Simpson’s dominance index calculated from total species abundances at each site reached its highest values at Sites 4 and 5 (Figure 6). This pattern reflects the pronounced dominance of *G. distigma* at these sites (Table 2). Dominance values at Sites 4 and 5 were at least two-fold higher than at most other sites and remained notably higher—by approximately 60%—even compared to Site 11. In contrast, the lowest dominance values were recorded at Sites 7, 8, and 9 (Figure 6).

### 3.5. Distribution of Drosophilidae Numbers in Post-Fire and Control Sites

**Figure 7:**
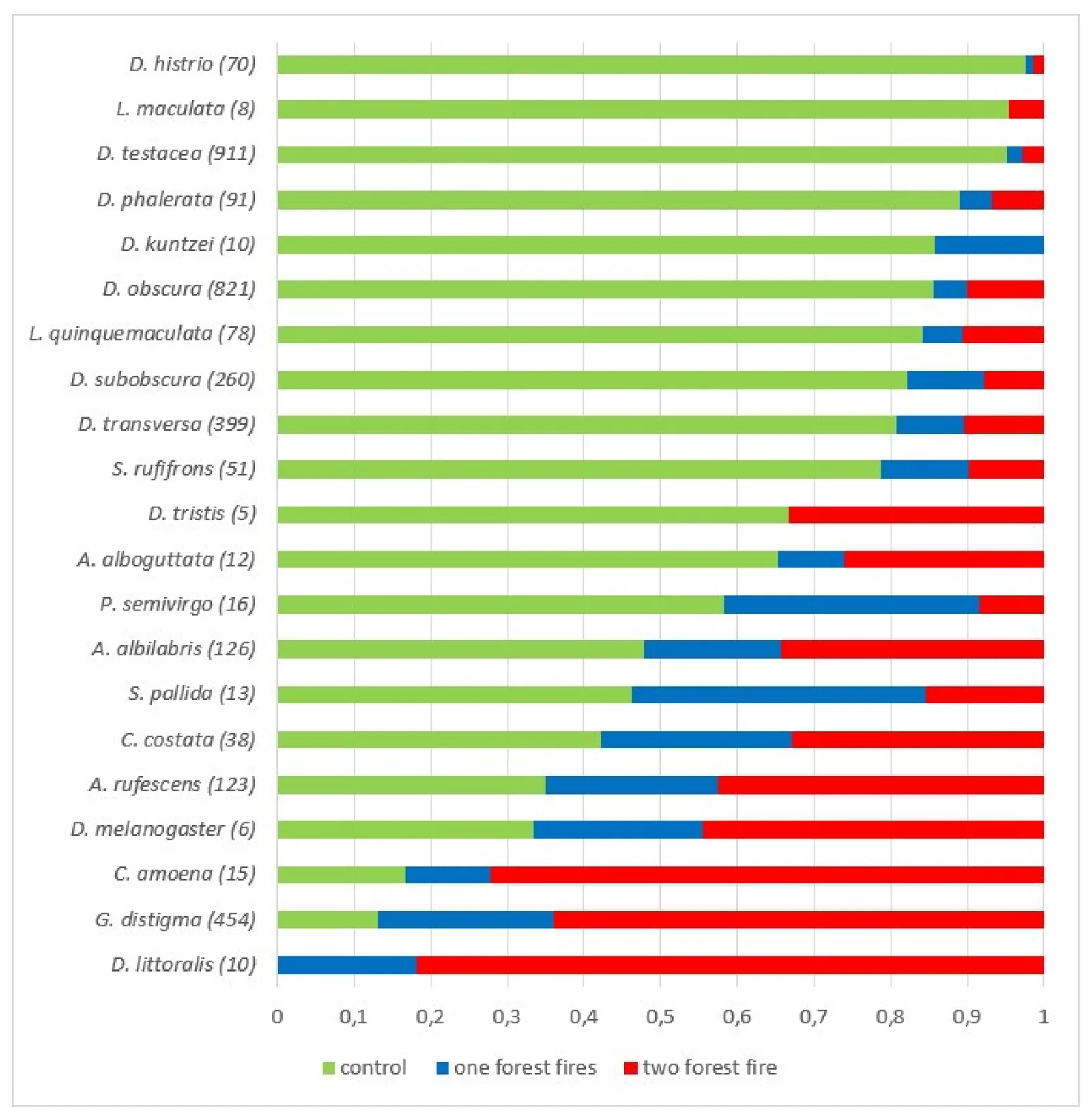
Relative representation of drosophilid species across post-fire and control sites over the entire sampling season. Data are averaged across sites with similar fire histories. For each site category, species proportions are calculated relative to the total number of individuals of that species across all samples; total abundances are given in parentheses. Species with fewer than five individuals in total were excluded.

Figure 7 shows the distribution of the relative abundances of drosophilid species across the sampling sites. Most species occurred at all site types, including both post-fire and unburned control sites. Species are ranked according to their total abundance at the control sites. *Drosophila histrio*, *Leucophenga maculata*, *D. testacea*, *D. phalerata*, *D. kuntzei*, *D. obscura*, *L. quinquemaculata*, *D. subobscura*, *D. transversa*, and *Scaptodrosophila rufifrons* occurred predominantly in assemblages at the control sites. By contrast, the combined relative abundances of *D. littoralis*, *Gitona distigma*, *Chymomyza amoena*, *D. melanogaster*, *Amiota rufescens*, *C. costata*, *Scaptomyza pallida*, and *A. albilabris* were higher at the post-fire sites. However, the low overall abundance of several of these species, together with substantial variation among sites, precluded their statistically robust identification as indicator species based on these distributional patterns alone.

**Figure 8:**
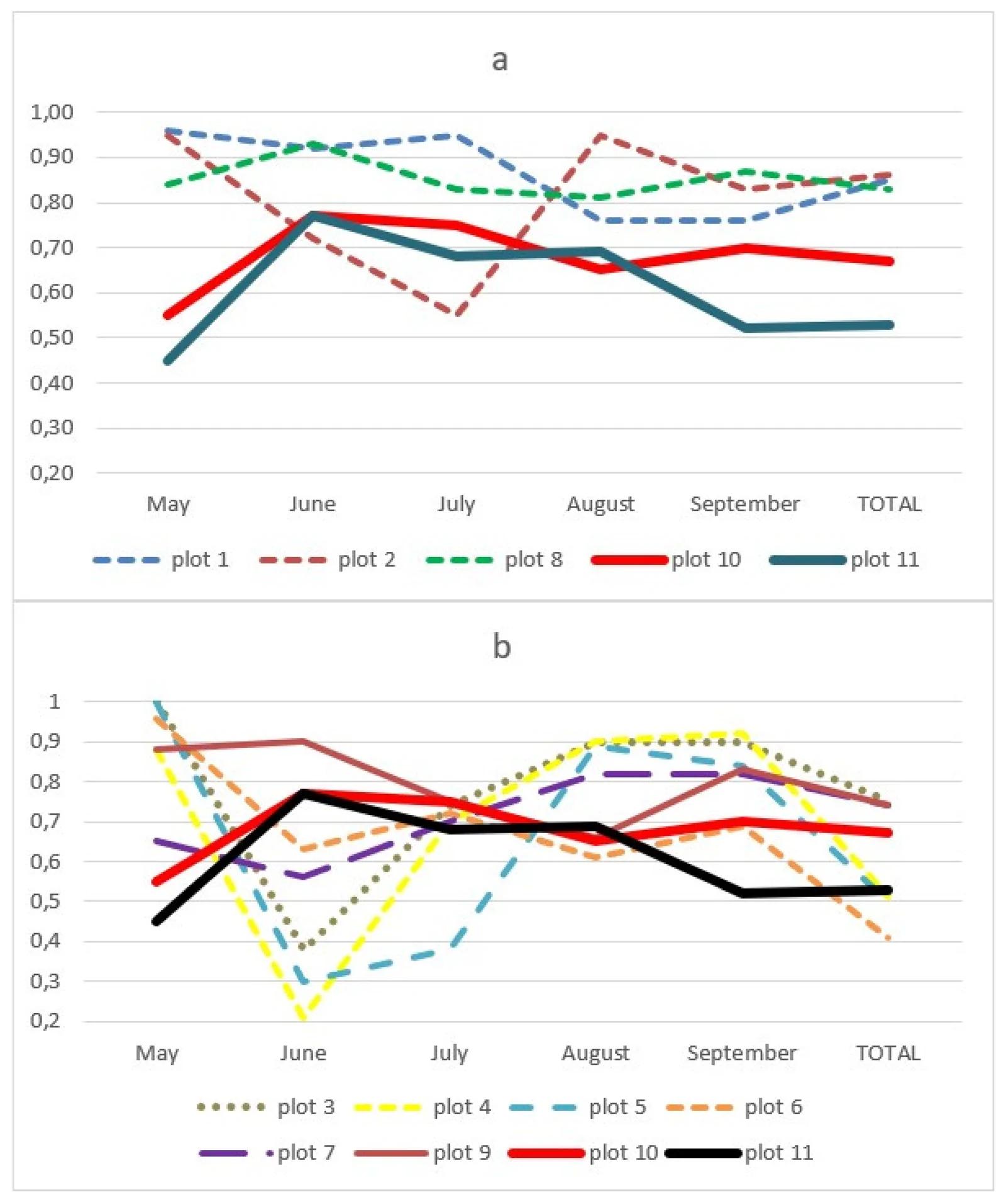
Pielou’s evenness (equitability) index of species distributions: (**a**) sites burned in 2010 and control sites; (**b**) sites burned twice (2010 and 2021) and control sites. Trends for control sites are shown by thick red and black lines.

Interestingly, the high total abundance of drosophilids at the control sites was accompanied by a pronouncedly uneven distribution of abundance among species (Figure 8).

In the control assemblages, the evenness index varied over the sampling season, with the lowest values being approximately 0.45. At the sites that burned in 2010, evenness was generally higher than at the control sites, except in July at Site 2 (Figure 8a). This pattern may indicate that drosophilid species occurred at similarly low abundances at fire-affected sites with limited food resources, whereas the control assemblages were characterized by the persistent dominance of several highly abundant species throughout the sampling season. At sites that burned twice, some assemblages exhibited substantial seasonal fluctuations in evenness (Figure 8b). At Sites 3, 4, 5, and 6, the relatively even distribution of species abundances observed in spring was replaced by pronounced dominance of one or several species in June. Abundances became more evenly distributed again in July and August, followed by renewed dominance of several species in September. At Sites 7 and 9, seasonal changes in evenness were similar to those observed at the control sites. The pronounced fluctuations in evenness at some post-fire sites may be associated with temporal changes in environmental conditions, including food availability. Sharp declines in evenness may reflect rapid increases in the abundance of one or several species when conditions become particularly favorable for them.

### 3.6. Drosophilid Species Associated with Unburned Control and Post-Fire Sites

Site-level IndVal.g analysis identified seven drosophilid species whose strongest association was with the unburned control group: *Drosophila histrio*, *D. testacea*, *D. phalerata*, *D. transversa*, *D. obscura*, *D. subobscura*, and *Leucophenga quinquemaculata* (Table 3). For each of these species, the exact unadjusted association test was nominally significant (*p* < 0.05), but none remained significant after Benjamini–Hochberg correction across the 30 drosophilid species (all P_FDR = 0.096). We therefore treat these taxa as candidate markers of intact forest conditions rather than as statistically validated indicator species.

Despite the lack of FDR-significant species-level associations, the quantitative contrasts were large. Mean seasonal abundance at the control sites was 8.1- to 74.3-fold higher than at fire-affected sites for the seven species listed in Table 3. The greatest differences were observed for *D. histrio* (33.0 versus 0.4 individuals per site) and *D. testacea* (407.0 versus 10.8 individuals per site). The other five species were approximately 8- to 15-fold more abundant at the unburned controls. These consistent abundance contrasts support their consideration as candidate markers of intact forest conditions, while the limited number of independent sites precludes formal validation in the present dataset.

*Gitona distigma* showed the opposite quantitative pattern. It occurred at all 11 sampling sites but its mean seasonal abundance was 47.7 individuals per fire-affected site compared with 12.5 per unburned control site, a 3.8-fold difference. Nevertheless, the complementary exact burned-versus-unburned IndVal.g analysis did not support formal indicator status (exact *p* = 0.0909; P_FDR = 0.273). Accordingly, *G. distigma* is treated here as a species showing a pronounced post-fire abundance pattern rather than as a statistically validated indicator.

Indicator-species analysis was also performed for the vegetation of the studied sites (Table 4).

In Group 1, comprising sites that burned in 2010, *Betula pendula*, particularly its young regeneration, was identified as an indicator species. *Calamagrostis epigejos* also showed an association approaching statistical significance. The dominance of young birch and *C. epigejos* is characteristic of clear-cuts, burned areas, and former agricultural land. These forest sites appear to represent a relatively advanced stage of post-fire recovery.

In Group 2, comprising sites that burned in both 2010 and 2021, the indicator species were *Epilobium angustifolium*, *Salix cinerea*, and *Pteridium pinetorum*. These species are among the early colonizers of burned areas and indicate the initial stages of post-fire vegetation recovery.

In Group 3, comprising control Sites 10 and 11, the indicator species were *Pinus sylvestris*, *Vaccinium vitis-idaea*, *Convallaria majalis*, and *Polygonatum odoratum*. These species are characteristic of mature bilberry–wood-sorrel pine forests, which typically have a closed canopy, a stable microclimate, and well-developed litter and soil profiles.

### 3.7. Seasonal Dynamics of Drosophilid Abundance at Post-Fire and Control Sites

**Figure 9:**
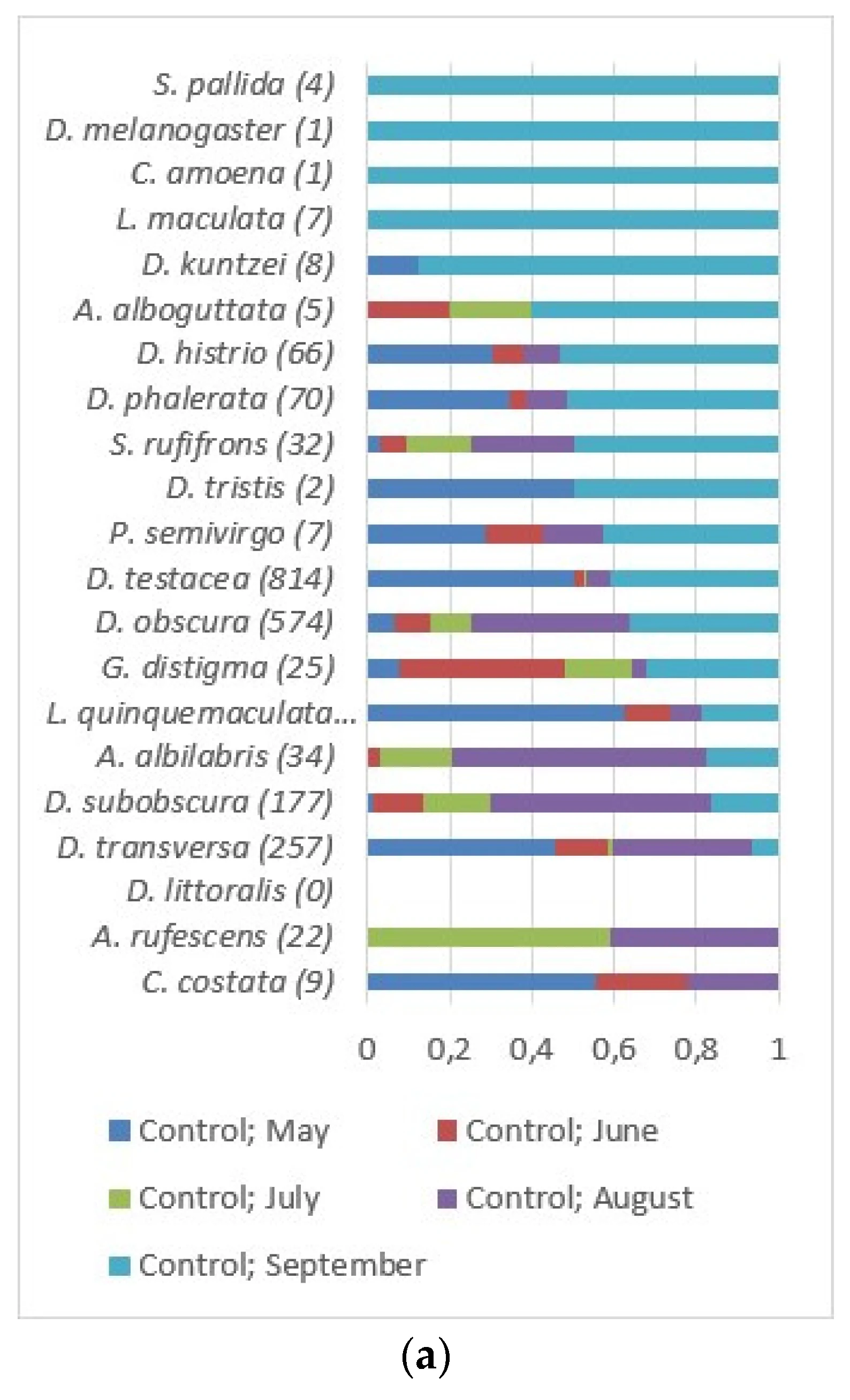

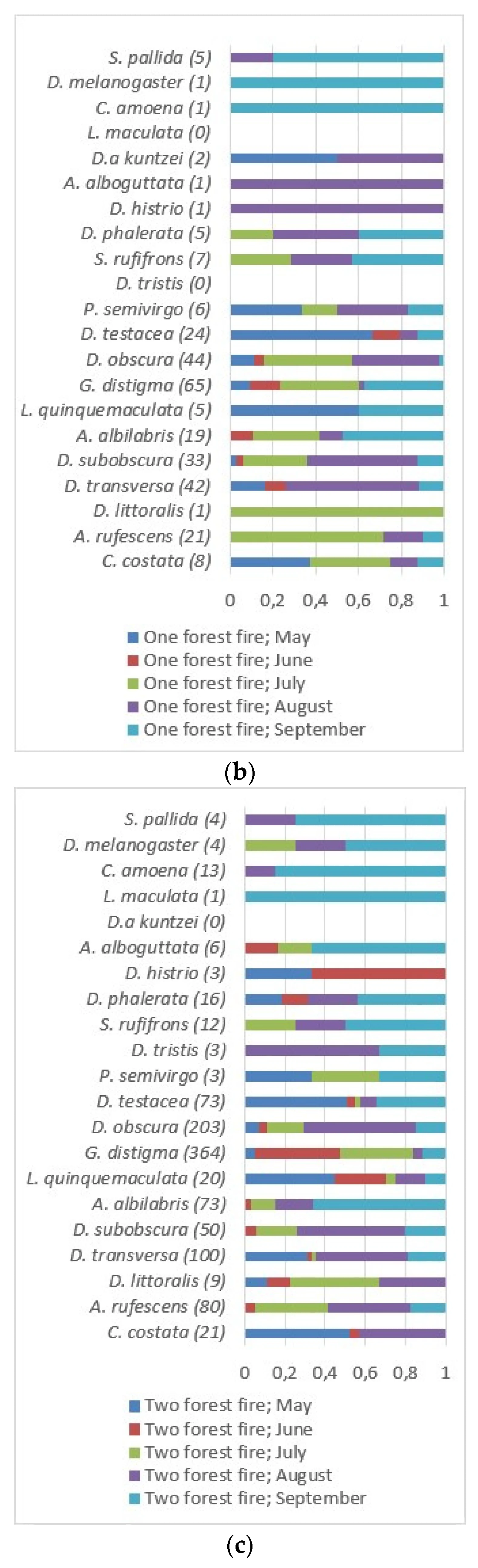
Monthly dynamics of drosophilid abundance at control and post-fire sites. Total numbers of drosophilids collected in each site category are given in parentheses. Species with fewer than five individuals in total were excluded. (**a**) Control forest sites (Sites 10 and 11); (**b**) sites burned once (Sites 1, 2, and 8); (**c**) sites burned twice (Sites 3–7 and 9).

Seasonal changes in drosophilid abundance were examined by month at post-fire and unburned control sites (Figure 9). In the assemblages of the unburned control forests, several species were recorded predominantly in autumn (Figure 9a), including *Scaptomyza pallida*, *Drosophila melanogaster*, *Chymomyza amoena*, *Leucophenga maculata*, and *D. kuntzei*. By contrast, *C. costata*, *Amiota rufescens*, *D. littoralis*, *D. transversa*, and *D. subobscura* were more frequently recorded from spring to mid-summer. Several post-fire sites showed pronounced summer abundance peaks, but the timing and magnitude of seasonal peaks varied substantially among individual sampling sites (Figure 9b,c).

Community composition showed a pronounced seasonal pattern after repeated observations from the same sampling sites were taken into account. In the analysis based on square-root-transformed species abundances, sampling month explained a substantial proportion of within-site temporal variation in assemblage structure (pseudo-F_4,40_ = 5.806, partial R^2^ = 0.367, *p* = 0.0002; Table 5). The seasonal effect was also evident in the relative-abundance sensitivity analysis (pseudo-F_4,40_ = 8.131, partial R^2^ = 0.448, *p* = 0.0002; Table S4), indicating that temporal changes involved shifts in species composition and were not caused solely by variation in total numbers of drosophilids captured.

Seasonal changes were more consistent for species richness than for total drosophilid abundance. Species richness differed significantly among months (Friedman χ^2^ = 18.373, *p* = 0.0010), whereas the corresponding repeated-measures test for total abundance was not significant (χ^2^ = 6.886, *p* = 0.142). Thus, although individual sampling sites differed in the timing and magnitude of abundance peaks, the composition and richness of drosophilid assemblages showed a consistent seasonal component across the study area.

## 4. Discussion

The first working hypothesis was supported: fire-affected drosophilid assemblages differed significantly from unburned controls after repeated seasonal observations were accounted for. The second hypothesis received partial support. Several species showed strong and biologically coherent habitat associations, but none of the drosophilid species-level IndVal.g associations remained significant after Benjamini–Hochberg correction when sampling site was treated as the independent spatial unit. In addition, the direct comparison of once- and twice-burned sites was not significant, indicating that the number of fires alone did not explain the observed heterogeneity within the post-fire landscape.

Wildfires are among the most powerful disturbance agents affecting boreal, temperate, and Mediterranean forests. They influence insect communities both directly, through mortality, and indirectly, through changes in habitat structure, microclimate, resource availability, and trophic interactions [17,41]. Post-fire recovery of insect assemblages is a complex, multifactorial process determined by fire severity and frequency, the size of the burned area, the time elapsed since fire, and species-specific biological traits, including dispersal capacity, life-cycle characteristics, and ecological requirements [42,43].

Fires can cause extensive insect mortality, particularly among species whose life stages occur in the forest litter, on the soil surface, or in tree canopies. Studies have shown that the abundance and species diversity of soil macrofauna during the first months after fire may be 1.5- to 5-fold lower than in unburned areas. Insects inhabiting deeper soil layers generally have higher post-fire survival than surface-dwelling species [44].

Post-fire recovery of insect fauna occurs through two principal pathways: (1) immigration from surrounding unburned forests and (2) survival in less severely burned areas that function as refugia or in deeper soil layers. Highly mobile insects, including many Diptera, Hymenoptera, and Lepidoptera, can colonize burned areas within the first weeks or months after fire [44,45]. Fire severity is a critical factor determining the rate and trajectory of insect-community recovery. In the boreal forests of Canada, low-severity burns in which some living trees remain support greater persistence of insect fauna than high-severity burns [46].

The main objective of the present study, conducted in 2022 in the Mordovia State Nature Reserve, was to investigate the abundance and species diversity of drosophilids at forest sites representing different stages of recovery after the severe fires of 2010 and after a repeated fire in 2021, in comparison with unburned control sites. Drosophilids constitute a useful model group among Diptera for studying the colonization of burned habitats. For example, species of the genus Drosophila dominated dipteran assemblages in recently burned areas in South Africa [47]. In southern California, *Drosophila pseudoobscura*, *D. persimilis*, *D. simulans*, and *D. melanogaster* were recorded within the burned area only 12 days after a catastrophic fire in the San Jacinto Mountains, several hundred meters from the fire perimeter [45].

A comprehensive study of insect-fauna recovery following the catastrophic fires of 2010 was also conducted in the Mordovia State Nature Reserve in 2019 [48]. That study showed that recovery proceeded through the formation of new, temporary biotic assemblages. Immediately after fire and during the first post-fire years, the insect fauna was dominated by xylobionts associated with dead wood, saprophages, and species favoring open, sun-exposed habitats. As the burned areas became colonized by grasses, shrubs, and young trees, these groups were gradually replaced by taxa associated with flowering plants, nectar resources, and dense understory vegetation, including anthophilous insects, wasps, predatory insects, and phytophages. Surface fires can increase forest heterogeneity by creating openings and deadwood habitats and may therefore promote biodiversity. By contrast, crown fires cause severe and long-lasting damage by destroying multiple structural layers of the ecosystem and require prolonged recovery. The previous study examined several insect orders collected from different burned sites. Among the 54 dipteran species recorded, Drosophilidae had the highest species richness, with 15 species [48]. The larvae of most of these drosophilids develop in decaying tissues beneath tree bark or in fermenting tree sap and can therefore be classified as xylosaprobionts. The most abundant drosophilids were *D. obscura* and *Gitona distigma*, with 224 and 138 individuals collected across all sites, respectively. Drosophila obscura reached its highest abundance at an unburned control site, although it was also well represented at burned sites containing abundant fallen birch, aspen, and alder wood. By contrast, *G. distigma* was represented by only isolated records at control sites with intact forest but reached high abundances at sites with a disturbed tree layer and abundant herbaceous vegetation, consistent with the development of its phytophagous larvae in inflorescences of Asteraceae [48].

Our study revealed substantial differences in the species composition, abundance, and seasonal dynamics of drosophilid assemblages between fire-affected and unburned control forests in the Mordovia State Nature Reserve. A total of 30 species belonging to nine genera were recorded, and most individuals, 61% of the total catch, were collected at the unburned control sites. This pattern is consistent with previous studies showing that fire can substantially reduce the total abundance of many insect groups through the loss or transformation of suitable habitats and food resources [19,27].

One of the principal findings was the clear separation of the sampling sites into two clusters based on species composition: post-fire Sites 1–9 and unburned control Sites 10 and 11. The high cophenetic correlation of the dendrogram (r = 0.941) indicates that this clustering closely represented the underlying Euclidean distance structure. The restricted permutation analysis independently confirmed a significant difference between fire-affected and unburned assemblages after seasonal variation was taken into account, and the contrast remained significant when total-catch differences were removed by using relative abundances. Notably, the passage of 12 years since the most recent fire at Sites 1, 2, and 8 did not result in complete convergence toward the assemblages observed at the control sites. At the same time, the direct comparison of once- and twice-burned sites was not significant, indicating that the number of fires alone did not explain the substantial heterogeneity observed within the post-fire landscape. Fire severity, moisture conditions, distance from the fire perimeter, and subsequent vegetation recovery are likely to contribute to this variation.

The unburned control sites supported assemblages characterized by very high abundances of several forest-associated drosophilids. Seven species—*D. histrio*, *D. testacea*, *D. phalerata*, *D. transversa*, *D. obscura*, *D. subobscura*, and *L. quinquemaculata*—showed their strongest site-level IndVal.g associations with the unburned group and were approximately 8- to 74-fold more abundant there than at fire-affected sites. Their unadjusted exact association tests were nominally significant, but none remained significant after correction for 30 species (P_FDR = 0.096). They should therefore be regarded as candidate markers of intact forest conditions rather than as statistically validated indicator species.

This strong numerical association is ecologically consistent with the greater availability of substrates required by many forest-dwelling drosophilids in intact forest. The control sites retained a well-developed litter layer, dead and decaying wood, fungi, and fermenting organic substrates that can support yeast- and bacteria-rich microhabitats. At fire-affected sites, especially where litter, bark, and woody substrates were strongly reduced, fewer suitable resources may have remained for larval development and recruitment of subsequent generations, and such habitats may also have been less attractive to adults. This interpretation is consistent with the known biology of many xylosaprobiont and mycetobiont drosophilids [49] and with the plant assemblage characteristic of mature forest at the control sites. However, microbial communities and larval breeding substrates were not quantified directly in this study, so this mechanism should be regarded as a biologically plausible interpretation rather than a demonstrated causal pathway.

*Gitona distigma* showed the reverse numerical tendency, with mean seasonal abundance almost four times higher at fire-affected sites than at the controls. The species was especially abundant at several strongly altered sites, consistent with the earlier observation in the reserve that it reached high abundance where the tree layer was disturbed and herbaceous vegetation was abundant [48]. Nevertheless, its corrected site-level association did not reach statistical significance, and we therefore do not regard *G. distigma* as a validated indicator of post-fire habitat in the present dataset. Its abundance pattern makes it a useful candidate for testing in larger, independently replicated post-fire datasets. It is also noteworthy that all synanthropic drosophilid species—*D. busckii*, *D. funebris*, *D. hydei*, *D. immigrans*, and *D. melanogaster*—were represented by only one to six individuals over the entire sampling season (Table 2). Their low abundances may indicate occasional dispersal into the forest rather than the presence of well-established populations within the reserve.

Seasonal changes in the Shannon diversity index also revealed differences in the temporal stability of drosophilid assemblages. At the control sites, diversity changed relatively gradually over the season, as might be expected in mature forest communities. At the post-fire sites, particularly Sites 1 and 2, which burned only once, diversity declined sharply in early summer and subsequently increased. These fluctuations may be associated with unstable availability of food resources, including fermenting plant substrates and fungi, as well as altered abiotic conditions, such as temperature and soil moisture, which may vary strongly over the season in disturbed habitats [50]. At twice-burned Sites 3–5, the relatively even distribution of species abundances observed in spring was replaced by pronounced dominance by one or two species in June. This pattern may reflect the ability of certain species to respond rapidly to temporarily favorable conditions in resource-limited post-fire habitats.

The persistence of the burned-versus-unburned difference in the relative-abundance analysis indicates that post-fire changes involved community composition and were not simply a consequence of lower total trap catches. This pattern is consistent with the known biology of drosophilids, many of which are xylosaprobionts or mycetobionts whose larvae develop beneath the bark of decaying trees, in fermenting tree sap, or in fungal substrates; a smaller number are phytophagous [49]. Fire can destroy the forest-litter layer and its associated fungi and can alter or consume bark and deadwood substrates, thereby removing microhabitats required for larval development. Subsequent vegetation recovery, including the establishment of dense *Betula pendula* regeneration at sites affected only by the 2010 fire and colonization by *Epilobium angustifolium* and *Pteridium pinetorum* at twice-burned sites, creates new ecological niches. Importantly, litter condition, deadwood availability, and vegetation structure are themselves components of post-fire habitat transformation rather than purely independent background covariates. These recovering habitats may therefore differ from mature forest in both resource availability and microclimatic conditions, which can contribute to the observed differences in drosophilid assemblages.

Several limitations of this study should be acknowledged. The study was observational rather than manipulative, with one standardized trap operated at each sampling site and only two unburned control sites; consequently, there was no within-site trap replication, and the sampling sites should not be interpreted as randomized experimental replicates of independent fire events. In particular, the sites affected by both the 2010 and 2021 fires were spatially separated sampling locations within a single 2021 fire-affected area. However, the control sites were selected from intact forest representing the same pre-fire mixed-forest habitat type, and before the fires the study area formed a continuous forest tract with high floristic similarity among the locations subsequently sampled. The restricted permutation analyses retained sampling site as the spatial observational unit and explicitly accounted for repeated monthly observations, but unmeasured site-specific environmental variation cannot be excluded. Beer-baited fermentation traps are effective for collecting adult drosophilids attracted to fermenting substrates and fungi but are selective, and habitat-dependent differences in temperature, humidity, and background odors could influence trap catches as well as local population abundance. Finally, sampling was conducted during a single field season, so interannual variation in weather, resource availability, and drosophilid population dynamics could not be separated from the patterns observed here. Multi-year studies with additional spatially independent fire-affected and control sites, within-site trap replication, and complementary methods—particularly Malaise traps, sweep netting, and emergence traps placed over decaying wood and fungal substrates—would provide a stronger basis for causal inference and biomonitoring applications.

## 5. Conclusions

Fire history and the resulting post-fire habitat transformation were associated with marked differences in drosophilid assemblages, including reduced overall catches and altered species composition relative to unburned forest. Most drosophilid individuals—2175 specimens, representing 61% of the total catch—were collected at the two unburned control sites. Community-level differences between fire-affected and unburned sites remained significant after repeated seasonal observations were accounted for and also when analyses were based on relative species abundances. By contrast, once- and twice-burned sites did not differ significantly when compared directly, indicating that variation within the post-fire landscape was not explained solely by the number of fires. Species diversity at some post-fire sites near the fire perimeter was comparable to that recorded at the controls. Seven drosophilid species showed strong quantitative associations with the unburned forest and may be regarded as candidate markers of intact forest conditions, but none remained statistically significant after FDR correction in the site-level analysis. *Gitona distigma* showed the opposite numerical abundance pattern but likewise did not meet the criteria for a validated indicator. Long-term studies incorporating additional spatial replication and complementary sampling approaches are therefore needed both to clarify mechanisms of post-fire succession and to validate species-level markers for biomonitoring in European Russia.

## Figures and Tables

**Table 1:** Summary characteristics of the 11 drosophilid sampling sites used for beer-baited trap sampling from May to September 2022 in the Mordovia State Nature Reserve, Republic of Mordovia, Central European Russia.

Site	Description
1	Burned only in 2010. Its tree layer consisted primarily of pine and birch, with dense regeneration of young birch. The shrub layer was dominated by raspberry (*Rubus* spp.), whereas the herb layer was sparse.
2	Also burned only in 2010 and was located 10 m outside the perimeter of the 2021 fire. Its vegetation was similar to that of Site 1.
3	Site 3 burned in both 2010 and 2021. In 2021, the site burned completely, including the standing dead trees and the shrub and herb layers. It was located 10 m inside the 2021 fire perimeter and 20 m from Site 2.
4	Also burned in both 2010 and 2021 and was completely burned in 2021, including its standing dead trees and shrub and herb layers. It was located 1000 m inside the 2021 fire perimeter.
5	It had a similar fire history and vegetation structure to Site 4 but was located 2000 m inside the fire perimeter.
6	Burned in both 2010 and 2021 and was located in a wet, low-lying area 1500 m inside the 2021 fire perimeter. This site experienced a low-severity burn in 2021. Some fallen trees and sparse birch regeneration that had developed after the 2010 fire remained, although the herb layer was almost completely burned in 2021.
7	Burned in both 2010 and 2021 and was located 10 m inside the 2021 fire perimeter. Birch trees, standing dead trees, shrubs, and herbaceous vegetation were only partially burned. At least half of the standing dead trees remained, together with dense stands of dead young birch following the fire.
8	Burned only in 2010. It was located 10 m outside the perimeter of the 2021 fire and 20 m from Site 7. The site contained abundant standing dead pine and birch trees, very dense regeneration of birch and aspen (*Populus tremula*), and a sparse herb layer.
9	Burned in both 2010 and 2021 and was located 10 m inside the 2021 fire perimeter. Birch trees, standing dead trees, shrubs, and herbaceous vegetation were partially burned. At least half of the standing dead trees remained, together with dense stands of dead young birch following the fire.
10	Control Site 10 was unaffected by either fire and was located 10 m outside the perimeters of the 2010 and 2021 fires. It consisted of old-growth mixed forest dominated by Pinus sylvestris and Betula spp., with Tilia cordata and Sorbus aucuparia occurring in the subcanopy. The forest-litter layer was well developed and deep, whereas the herb layer was sparse.
11	Control Site 11 was also unaffected by fire and was located 500 m from the perimeters of the 2010 and 2021 fires. Its vegetation composition and vertical structure were similar to those of Site 10.

**Table 2:** Total abundance and species diversity of *Drosophilidae* (*Diptera*) collected using beer-baited fermentation traps at 11 study sites in the Mordovia State Nature Reserve, Republic of Mordovia, Central European Russia, during May–September 2022. Sites 1–9 are post-fire habitats; Sites 10 and 11 are unburned controls.

Species	Site 1	Site 2	Site 3	Site 4	Site 5	Site 6	Site 7	Site 8	Site 9	Site 10	Site 11	Total
Subfamily Steganinae												
*Amiota albilabris* (Roth in Zetterstedt, 1860)	0	3	2	3	4	37	18	16	9	32	2	126
*Amiota alboguttata* (Wahlberg, 1839)	1	0	1	0	1	1	1	0	2	5	0	12
*Amiota rufescens* (Oldenberg, 1914)	0	2	2	11	8	34	17	19	8	20	2	123
*Amiota subtusradiata* (Duda, 1934)	0	0	0	0	0	1	0	0	0	0	0	1
*Gitona distigma* (Meigen, 1830)	14	21	30	100	98	56	73	30	7	23	2	454
*Leucophenga maculata* (Dufour, 1839)	0	0	0	0	0	0	0	0	1	6	1	8
*Leucophenga quinquemaculata* (Strobl, 1893)	1	1	1	0	0	2	3	3	14	31	22	78
*Phortica semivirgo* (Maca, 1977)	2	2	0	0	1	0	2	2	0	2	5	16
Subfamily Drosophilinae												
*Chymomyza amoena* (Loew, 1862)	0	0	4	1	5	0	2	1	1	0	1	15
*Chymomyza caudatula* (Oldenberg, 1914)	0	0	0	0	0	1	2	0	0	0	0	3
*Chymomyza costata* (Zetterstedt, 1838)	0	1	1	1	0	4	6	7	9	4	5	38
*Chymomyza fuscimana* (Zetterstedt, 1838)	0	0	1	0	0	0	0	0	1	0	1	3
*Drosophila busckii* (Coquillett, 1901)	0	0	1	0	0	0	0	0	0	0	0	1
*Drosophila funebris* (Fabricius, 1787)	0	0	0	0	0	1	0	0	0	0	0	1
*Drosophila histrio* (Meigen, 1830)	0	1	0	0	0	0	2	0	1	16	50	70
*Drosophila hydei* (Sturtevant, 1921)	0	0	2	0	0	0	0	0	0	0	0	2
*Drosophila immigrans* (Sturtevant, 1921)	0	0	0	0	0	0	0	0	1	1	2	4
*Drosophila kuntzei* (Duda, 1924)	0	0	0	0	0	0	0	2	0	0	8	10
*Drosophila littoralis* (Meigen, 1830)	0	0	2	1	2	2	2	1	0	0	0	10
*Drosophila melanogaster* (Meigen, 1830)	0	0	1	0	0	2	1	1	0	1	0	6
*Drosophila obscura* (Fallén, 1823)	10	4	10	21	10	77	46	30	39	282	292	821
*Drosophila phalerata* (Meigen, 1830)	0	0	1	1	0	3	7	5	4	16	54	91
*Drosophila subobscura* (Collin in Gordon, 1936)	10	3	1	5	3	19	9	20	13	113	64	260
*Drosophila testacea* (von Roser, 1840)	6	1	0	2	1	3	13	17	54	232	582	911
*Drosophila transversa* (Fallén, 1823)	15	2	2	1	3	6	32	25	56	178	79	399
*Drosophila tristis* (Fallén, 1823)	0	0	0	0	0	1	1	0	1	2	0	5
*Hirtodrosophila confusa* (Staeger, 1844)	0	0	0	0	0	0	0	0	0	0	2	2
*Hirtodrosophila toyohiokadai* (Sidorenko, 1990)	0	0	0	0	0	0	0	2	0	0	1	3
*Scaptodrosophila rufifrons* (Loew, 1873)	2	1	2	0	1	0	5	4	4	14	18	51
*Scaptomyza pallida* (Zetterstedt, 1847)	1	3	0	0	0	0	1	1	3	3	1	13
Total individuals (number of species)	62 (10)	45 (13)	64 (17 )	147 (11)	137 (12)	250 (17)	243 (20)	186 (18)	228 (19)	981 (19)	1194 (21)	3537 (30)

**Table 3:** Drosophilid species showing the strongest associations with unburned control forest sites in the Mordovia State Nature Reserve, Republic of Mordovia, Central European Russia, based on seasonal totals from beer-baited trap catches collected May–September 2022.

Species	IndVal.g (%)	Mean Abundance Control	Mean Abundance Fire-Affected	Control/ Fire-Affected	Exact *p*	P_FDR (BH)
*Drosophila histrio*	98.8	33.0	0.4	74.3	0.0206	0.0963
*Drosophila testacea*	97.6	407.0	10.8	37.8	0.0340	0.0963
*Drosophila phalerata*	94.3	35.0	2.3	15.0	0.0184	0.0963
*Drosophila transversa*	89.9	128.5	15.8	8.1	0.0385	0.0963
*Drosophila obscura*	92.5	287.0	27.4	10.5	0.0050	0.0963
*Drosophila subobscura*	90.6	88.5	9.2	9.6	0.0201	0.0963
*Leucophenga quinquemaculata*	91.7	26.5	2.8	9.5	0.0385	0.0963

IndVal.g (%), group-equalized association statistic reported as 100 × sqrt(A × B), where A measures specificity to the target group and B measures fidelity within that group. Mean abundances are seasonal totals per sampling site. Exact *p* values are from all 4620 allocations of the 11 sampling sites among the three fire-history groups (unburned, n = 2; burned once, n = 3; burned twice, n = 6), with associations restricted to individual groups. P_FDR (BH), Benjamini–Hochberg-adjusted *p* value across 30 drosophilid species. All species listed in the table were most strongly associated with the unburned control group; none remained significant at FDR = 0.05.

**Table 4:** Indicator plant species for control (unburned) and post-fire forest sites in the Mordovia State Nature Reserve, Republic of Mordovia, Central European Russia, sampled during the 2022 field season.

Species	Indicator Group	IndVal	p.raw (9999 Perms)	p.FDR (BH)
*Pinus sylvestris*	Group 3	100.0	<0.0001	0.0038
*Vaccinium vitis-idaea*	Group 3	94.7	0.0001	0.0038
*Convallaria majalis*	Group 3	93.6	0.0001	0.0038
*Polygonatum odoratum*	Group 3	72.2	0.002	0.015
*Epilobium angustifolium*	Group 2	77.8	0.001	0.0095
*Salix cinerea*	Group 2	66.7	0.006	0.025
*Pteridium pinetorum*	Group 2	62.2	0.009	0.033
*Betula pendula*	Group 1	67.7	0.003	0.019
*Calamagrostis epigejos*	Group 1	57.3	0.018	0.057

IndVal, indicator value index measuring the association of a species with a habitat type (maximum value 100); p.raw, *p*-values from permutation tests (9999 permutations); p.FDR (BH), *p*-values adjusted for multiple testing using the Benjamini–Hochberg procedure. Groups 1 and 2, post-fire forest sites burned once (Sites 1, 2, and 8) and twice (Sites 3–7 and 9), respectively; Group 3, unburned control sites (Sites 10 and 11).

**Table 5:** Restricted permutation analyses of variation in drosophilid community composition associated with season and fire history in the Mordovia State Nature Reserve, Republic of Mordovia, Central European Russia, based on monthly beer-baited trap catches collected May–September 2022.

Effect/Comparison	Data Representation	Blocking/Adjustment	Pseudo-F	df	Partial R^2^	*p*
Sampling month	Square-root abundance	Site	5.806	4, 40	0.367	0.0002
Burned vs. unburned	Square-root abundance	Month	11.597	1, 49	0.191	0.0182
Burned vs. unburned	Relative abundance	Month	10.225	1, 49	0.173	0.0182
Fire history: 0, 1, or 2 fires	Square-root abundance	Month	6.896	2, 48	0.223	0.0130
Fire history: 0, 1, or 2 fires	Relative abundance	Month	5.899	2, 48	0.197	0.0286
Once- vs. twice-burned sites	Square-root abundance	Month	1.818	1, 39	0.045	0.333
Once- vs. twice-burned sites	Relative abundance	Month	1.408	1, 39	0.035	0.512

Blocking/adjustment specifies the factor used to constrain or adjust the permutation scheme for the corresponding effect. For sampling month, site was used as a blocking factor and month labels were permuted only within sites. For between-site fire-history comparisons, Month was included as an adjustment factor and fire-history labels were permuted only among whole sampling sites, with all monthly observations from the same site kept together. Square-root abundance, square-root-transformed species counts; Relative abundance, species proportions within each site × month sample; pseudo-F, distance-based pseudo-F statistic; partial R^2^, effect size; *p*, exact permutation *p* value for between-site comparisons and restricted permutation *p* value for the seasonal test.

## Data Availability

The original contributions presented in this study are included in the article/Supplementary Materials. Further inquiries can be directed to the corresponding author.

## Supplementary Materials

The following supporting information can be downloaded at: https://www.mdpi.com/article/10.3390/insects17090892/s1, Table S1: Braun–Blanquet vegetation cover–abundance data recorded at the 11 sampling sites in 2022; Table S2: Descriptive statistics of seasonal site-level drosophilid abundances across the 11 sampling sites; Table S3: Monthly abundance of Drosophilidae at all sampling sites, May–September 2022; Table S4: Complete results of restricted permutation analyses and sensitivity analyses of drosophilid community composition.
