# Supplementary material for: Diversity and Abundance of Drosophilidae (Diptera) in Post-Fire Forest Ecosystems of Central European Russia
Source: Insects. 2026 Aug 25;17(9):892. doi: 10.3390/insects17090892 (PMC13607579; doi:10.3390/insects17090892)
Supplement: Supplementary file 1 [file insects-17-00892-s001.zip › insects-4483936-supplementary/insects-4483936_Supplementary_Materials_after_revision.pdf]

Supplementary Materials

Diversity and Abundance of Drosophilidae (Diptera) In Post-Fire Forest Ecosystems of Central European Russia

Gornostaev et al.

Table S1. Braun–Blanquet vegetation cover–abundance data recorded at the 11 sampling sites in 2022.

Layer	Species	Site 1	Site 2	Site 3	Site 4	Site 5	Site 6	Site 7	Site 8	Site 9	Site 10	Site 11
Tree layer	*Pinus sylvestris*	0	0	0	0	0	0	0	0	0	65	65
Tree layer	*Betula pendula*	0	0	0	0	0	1	0	0	0	5	0
Shrub-sapling layer	*Salix cinerea*	0	0	0	3	3	8	1	0	2	0	0
Shrub-sapling layer	*Betula pendula*	60	22	3	5	10	5	8	25	12	3	5
Shrub-sapling layer	*Pinus sylvestris*	1	7	1	3	3	6	5	3	5	5	2
Shrub-sapling layer	*Populus tremula*	5	0	0	5	5	0	1	5	3	0	2
Shrub-sapling layer	*Picea abies*	0	0	0	0	0	0	0	0	0	0	4
Shrub-sapling layer	*Frangula alnus*	0	0	0	0	0	0	0	0	0	5	3
Shrub-sapling layer	*Rubus idaeus*	0	0	0	0	0	0	1	3	3	1	0
Shrub-sapling layer	*Sambucus racemosa*	0	0	0	0	0	0	0	2	0	0	0
Shrub-sapling layer	*Sorbus aucuparia*	1	1	1	0	0	2	1	0	2	5	3
Shrub-sapling layer	*Chamaecytisus ruthenicus*	5	8	0	3	3	2	1	0	0	2	5
Ground layer	*Calamagrostis epigejos*	45	55	30	25	13	0	10	25	7	8	0
Ground layer	*Epilobium angustifolium*	4	1	5	2	6	2	20	7	7	0	0
Ground layer	*Rubus saxatilis*	5	2	0	0	0	3	1	0	1	7	5
Ground layer	*Solidago canadensis*	2	0	0	0	0	0	0	0	2	0	0
Ground layer	*Geranium sanguineum*	0	0	0	0	0	1	0	0	0	1	0
Ground layer	*Carex digitata*	0	0	0	0	0	3	2	0	2	8	2
Ground layer	*Pteridium pinetorum*	0	0	0	0	0	5	15	5	50	0	0
Ground layer	*Convallaria majalis*	0	0	0	0	0	2	2	0	1	28	45
Ground layer	*Vaccinium vitis-idaea*	0	0	0	0	0	0	0	0	1	8	10
Ground layer	*Polygonatum odoratum*	5	0	0	0	0	0	0	0	0	5	8
Ground layer	*Agrostis gigantea*	0	0	4	0	1	0	0	0	0	3	5
Ground layer	*Campanula rotundifolia*	2	0	0	0	0	0	0	1	0	3	0
Ground layer	*Hieracium umbellatum*	5	0	1	2	6	4	0	0	0	1	0
Ground layer	*Achillea millefolium*	1	0	1	0	0	0	0	0	0	0	0
Ground layer	*Erigeron canadensis*	0	5	20	0	4	0	8	10	2	0	0
Ground layer	*Rumex acetosella*	1	2	0	5	3	0	0	0	0	0	0
Ground layer	*Erigeron annuus*	0	1	0	0	0	0	0	0	0	0	0
Ground layer	*Digitaria ishaemum*	0	0	0	0	2	0	0	0	0	0	0
Ground layer	*Solidago virgaurea*	0	0	0	0	0	5	0	0	0	5	0
Ground layer	*Hypericum perforatum*	0	0	0	0	0	0	0	4	0	0	0
Ground layer	*Calamagrostis arundinacea*	0	0	0	0	0	0	0	0	0	0	10
Ground layer	*Dryopteris carthusiana*	0	0	0	0	0	0	0	0	0	1	1
Ground layer	*Melampyrum pratense*	0	0	0	0	0	0	0	0	0	5	6
Ground layer	*Berteroa incana*	0	0	1	0	0	0	0	0	0	0	0
Ground layer	*Molinia coerulea*	0	0	0	0	0	6	0	0	0	0	0
Ground layer	*Anthoxanthum odoratum*	0	0	0	0	0	2	0	0	0	0	0

Table S2. Descriptive statistics of seasonal site-level drosophilid abundances across the 11 sampling sites.

Species	Mean	Median	SD	Q1	Q3	Min	Max	Skewness	Excess kurtosis
Amiota albilabris	11.45	4.00	12.86	2.50	17.00	0	37	1.20	0.22
Amiota alboguttata	1.09	1.00	1.45	0.00	1.00	0	5	2.24	5.89
Amiota rufescens	11.18	8.00	10.45	2.00	18.00	0	34	1.01	0.74
Amiota subtusradiata	0.09	0.00	0.30	0.00	0.00	0	1	3.32	11.00
Gitona distigma	41.27	30.00	35.14	17.50	64.50	2	100	0.79	-0.81
Leucophenga maculata	0.73	0.00	1.79	0.00	0.50	0	6	3.04	9.56
Leucophenga quinquemaculata	7.09	2.00	10.55	1.00	8.50	0	31	1.63	1.59
Phortica semivirgo	1.45	2.00	1.51	0.00	2.00	0	5	1.18	2.09
Chymomyza amoena	1.36	1.00	1.69	0.00	1.50	0	5	1.43	1.17
Chymomyza caudatula	0.27	0.00	0.65	0.00	0.00	0	2	2.42	5.51
Chymomyza costata	3.45	4.00	3.08	1.00	5.50	0	9	0.46	-1.01
Chymomyza fuscimana	0.27	0.00	0.47	0.00	0.50	0	1	1.19	-0.76
Drosophila busckii	0.09	0.00	0.30	0.00	0.00	0	1	3.32	11.00
Drosophila funebris	0.09	0.00	0.30	0.00	0.00	0	1	3.32	11.00
Drosophila histrio	6.36	0.00	15.22	0.00	1.50	0	50	2.84	8.29
Drosophila hydei	0.18	0.00	0.60	0.00	0.00	0	2	3.32	11.00
Drosophila immigrans	0.36	0.00	0.67	0.00	0.50	0	2	1.80	2.61
Drosophila kuntzei	0.91	0.00	2.43	0.00	0.00	0	8	3.00	9.23
Drosophila littoralis	0.91	1.00	0.94	0.00	2.00	0	2	0.21	-2.07
Drosophila melanogaster	0.55	0.00	0.69	0.00	1.00	0	2	0.93	0.08
Drosophila obscura	74.64	30.00	107.11	10.00	61.50	4	292	1.76	1.65
Drosophila phalerata	8.27	3.00	15.88	0.50	6.00	0	54	2.85	8.52
Drosophila subobscura	23.64	10.00	34.46	4.00	19.50	1	113	2.20	4.58
Drosophila testacea	82.82	6.00	179.03	1.50	35.50	0	582	2.64	7.06
Drosophila transversa	36.27	15.00	53.40	2.50	44.00	1	178	2.19	5.16
Drosophila tristis	0.45	0.00	0.69	0.00	1.00	0	2	1.32	0.98
Hirtodrosophila confusa	0.18	0.00	0.60	0.00	0.00	0	2	3.32	11.00
Hirtodrosophila toyohiokadai	0.27	0.00	0.65	0.00	0.00	0	2	2.42	5.51
Scaptodrosophila rufifrons	4.64	2.00	5.92	1.00	4.50	0	18	1.69	1.94
Scaptomyza pallida	1.18	1.00	1.25	0.00	2.00	0	3	0.71	-1.12

Note. Statistics were calculated from seasonal totals for each of the 11 sampling sites (n = 11 independent spatial units). SD, sample standard deviation; Q1 and Q3, first and third quartiles; excess kurtosis is reported relative to the normal distribution.

Table S3. Monthly abundance of Drosophilidae at all sampling sites, May–September 2022.

Table layout. The table is divided into four continuation panels for readability. Each site contributes five monthly columns (May–September); the final row gives the total number of individuals in each site-month sample.

Sites 1-3.

Species	S1 May	S1 Jun	S1 Jul	S1 Aug	S1 Sep	S2 May	S2 Jun	S2 Jul	S2 Aug	S2 Sep	S3 May	S3 Jun	S3 Jul	S3 Aug	S3 Sep
Amiota albilabris	0	0	0	0	0	0	1	0	0	2	0	0	1	1	0
Amiota alboguttata	0	0	0	1	0	0	0	0	0	0	0	1	0	0	0
Amiota rufescens	0	0	0	0	0	0	0	0	2	0	0	0	1	1	0
Amiota subtusradiata	0	0	0	0	0	0	0	0	0	0	0	0	0	0	0
Gitona distigma	1	2	1	1	9	0	4	9	1	7	1	14	10	1	4
Leucophenga maculata	0	0	0	0	0	0	0	0	0	0	0	0	0	0	0
Leucophenga quinquemaculata	1	0	0	0	0	0	0	0	0	1	1	0	0	0	0
Phortica semivirgo	0	0	1	1	0	2	0	0	0	0	0	0	0	0	0
Chymomyza amoena	0	0	0	0	0	0	0	0	0	0	0	0	0	1	3
Chymomyza caudatula	0	0	0	0	0	0	0	0	0	0	0	0	0	0	0
Chymomyza costata	0	0	0	0	0	0	0	0	1	0	0	0	0	1	0
Chymomyza fuscimana	0	0	0	0	0	0	0	0	0	0	0	0	0	0	1
Drosophila busckii	0	0	0	0	0	0	0	0	0	0	0	0	0	1	0
Drosophila funebris	0	0	0	0	0	0	0	0	0	0	0	0	0	0	0
Drosophila histrio	0	0	0	0	0	0	0	0	1	0	0	0	0	0	0
Drosophila hydei	0	0	0	0	0	0	0	0	0	0	0	0	0	0	2
Drosophila immigrans	0	0	0	0	0	0	0	0	0	0	0	0	0	0	0
Drosophila kuntzei	0	0	0	0	0	0	0	0	0	0	0	0	0	0	0
Drosophila littoralis	0	0	0	0	0	0	0	0	0	0	0	0	1	1	0
Drosophila melanogaster	0	0	0	0	0	0	0	0	0	0	0	0	0	0	1
Drosophila obscura	2	0	2	5	1	0	0	1	3	0	0	0	5	5	0
Drosophila phalerata	0	0	0	0	0	0	0	0	0	0	0	0	0	1	0
Drosophila subobscura	0	0	3	5	2	0	0	1	2	0	0	0	1	0	0
Drosophila testacea	1	1	0	2	2	1	0	0	0	0	0	0	0	0	0
Drosophila transversa	0	0	0	14	1	1	0	0	1	0	1	0	0	1	0
Drosophila tristis	0	0	0	0	0	0	0	0	0	0	0	0	0	0	0
Hirtodrosophila confusa	0	0	0	0	0	0	0	0	0	0	0	0	0	0	0
Hirtodrosophila toyohiokadai	0	0	0	0	0	0	0	0	0	0	0	0	0	0	0
Scaptodrosophila rufifrons	0	0	1	1	0	0	0	0	0	1	0	0	1	0	1
Scaptomyza pallida	0	0	0	1	0	0	0	0	0	3	0	0	0	0	0
**Total individuals**	**5**	**3**	**8**	**31**	**15**	**4**	**5**	**11**	**11**	**14**	**3**	**15**	**20**	**14**	**12**

Table S3 (continued). Sites 4-6.

Species	S4 May	S4 Jun	S4 Jul	S4 Aug	S4 Sep	S5 May	S5 Jun	S5 Jul	S5 Aug	S5 Sep	S6 May	S6 Jun	S6 Jul	S6 Aug	S6 Sep
Amiota albilabris	0	0	0	3	0	0	0	1	1	2	0	2	5	3	27
Amiota alboguttata	0	0	0	0	0	0	0	0	0	1	0	0	0	0	1
Amiota rufescens	0	1	4	6	0	0	0	5	3	0	0	2	16	13	3
Amiota subtusradiata	0	0	0	0	0	0	0	0	0	0	0	0	0	0	1
Gitona distigma	4	69	20	5	2	1	35	59	2	1	0	18	20	3	15
Leucophenga maculata	0	0	0	0	0	0	0	0	0	0	0	0	0	0	0
Leucophenga quinquemaculata	0	0	0	0	0	0	0	0	0	0	1	0	0	1	0
Phortica semivirgo	0	0	0	0	0	1	0	0	0	0	0	0	0	0	0
Chymomyza amoena	0	0	0	1	0	0	0	0	0	5	0	0	0	0	0
Chymomyza caudatula	0	0	0	0	0	0	0	0	0	0	0	0	0	1	0
Chymomyza costata	1	0	0	0	0	0	0	0	0	0	1	0	0	3	0
Chymomyza fuscimana	0	0	0	0	0	0	0	0	0	0	0	0	0	0	0
Drosophila busckii	0	0	0	0	0	0	0	0	0	0	0	0	0	0	0
Drosophila funebris	0	0	0	0	0	0	0	0	0	0	0	0	0	1	0
Drosophila histrio	0	0	0	0	0	0	0	0	0	0	0	0	0	0	0
Drosophila hydei	0	0	0	0	0	0	0	0	0	0	0	0	0	0	0
Drosophila immigrans	0	0	0	0	0	0	0	0	0	0	0	0	0	0	0
Drosophila kuntzei	0	0	0	0	0	0	0	0	0	0	0	0	0	0	0
Drosophila littoralis	0	1	0	0	0	0	0	2	0	0	0	0	1	1	0
Drosophila melanogaster	0	0	0	0	0	0	0	0	0	0	0	0	0	1	1
Drosophila obscura	1	2	9	8	1	0	2	3	5	0	1	1	4	65	6
Drosophila phalerata	1	0	0	0	0	0	0	0	0	0	0	1	0	1	1
Drosophila subobscura	0	1	1	3	0	0	1	1	1	0	0	1	1	16	1
Drosophila testacea	1	0	0	1	0	1	0	0	0	0	0	1	1	1	0
Drosophila transversa	0	0	1	0	0	1	0	1	0	1	2	1	0	2	1
Drosophila tristis	0	0	0	0	0	0	0	0	0	0	0	0	0	1	0
Hirtodrosophila confusa	0	0	0	0	0	0	0	0	0	0	0	0	0	0	0
Hirtodrosophila toyohiokadai	0	0	0	0	0	0	0	0	0	0	0	0	0	0	0
Scaptodrosophila rufifrons	0	0	0	0	0	0	0	0	1	0	0	0	0	0	0
Scaptomyza pallida	0	0	0	0	0	0	0	0	0	0	0	0	0	0	0
**Total individuals**	**8**	**74**	**35**	**27**	**3**	**4**	**38**	**72**	**13**	**10**	**5**	**27**	**48**	**113**	**57**

Table S3 (continued). Sites 7-9.

Species	S7 May	S7 Jun	S7 Jul	S7 Aug	S7 Sep	S8 May	S8 Jun	S8 Jul	S8 Aug	S8 Sep	S9 May	S9 Jun	S9 Jul	S9 Aug	S9 Sep
Amiota albilabris	0	0	2	4	12	0	1	6	2	7	0	0	0	2	7
Amiota alboguttata	0	0	1	0	0	0	0	0	0	0	0	0	0	0	2
Amiota rufescens	0	0	2	9	6	0	0	15	2	2	0	1	1	1	5
Amiota subtusradiata	0	0	0	0	0	0	0	0	0	0	0	0	0	0	0
Gitona distigma	13	16	23	5	16	5	3	14	0	8	0	1	1	0	5
Leucophenga maculata	0	0	0	0	0	0	0	0	0	0	0	0	0	0	1
Leucophenga quinquemaculata	0	2	1	0	0	2	0	0	0	1	7	3	0	2	2
Phortica semivirgo	0	0	1	0	1	0	0	0	1	1	0	0	0	0	0
Chymomyza amoena	0	0	0	0	2	0	0	0	0	1	0	0	0	0	1
Chymomyza caudatula	1	0	0	0	1	0	0	0	0	0	0	0	0	0	0
Chymomyza costata	1	0	0	5	0	3	0	3	0	1	8	1	0	0	0
Chymomyza fuscimana	0	0	0	0	0	0	0	0	0	0	0	0	0	0	1
Drosophila busckii	0	0	0	0	0	0	0	0	0	0	0	0	0	0	0
Drosophila funebris	0	0	0	0	0	0	0	0	0	0	0	0	0	0	0
Drosophila histrio	1	1	0	0	0	0	0	0	0	0	0	1	0	0	0
Drosophila hydei	0	0	0	0	0	0	0	0	0	0	0	0	0	0	0
Drosophila immigrans	0	0	0	0	0	0	0	0	0	0	0	0	0	0	1
Drosophila kuntzei	0	0	0	0	0	1	0	0	1	0	0	0	0	0	0
Drosophila littoralis	1	0	0	1	0	0	0	1	0	0	0	0	0	0	0
Drosophila melanogaster	0	0	1	0	0	0	0	0	0	1	0	0	0	0	0
Drosophila obscura	7	0	9	23	7	3	2	15	10	0	5	4	7	7	16
Drosophila phalerata	2	1	0	2	2	0	0	1	2	2	0	0	0	0	4
Drosophila subobscura	0	0	4	3	2	1	1	6	10	2	0	0	2	4	7
Drosophila testacea	7	1	1	4	0	14	2	0	0	1	28	1	0	0	25
Drosophila transversa	12	1	0	16	3	6	4	0	11	4	15	0	0	27	14
Drosophila tristis	0	0	0	1	0	0	0	0	0	0	0	0	0	0	1
Hirtodrosophila confusa	0	0	0	0	0	0	0	0	0	0	0	0	0	0	0
Hirtodrosophila toyohiokadai	0	0	0	0	0	2	0	0	0	0	0	0	0	0	0
Scaptodrosophila rufifrons	0	0	2	2	1	0	0	1	1	2	0	0	0	0	4
Scaptomyza pallida	0	0	0	1	0	0	0	0	0	1	0	0	0	0	3
**Total individuals**	**45**	**22**	**47**	**76**	**53**	**37**	**13**	**62**	**40**	**34**	**63**	**12**	**11**	**43**	**99**

Table S3 (continued). Sites 10-11.

Species	S10 May	S10 Jun	S10 Jul	S10 Aug	S10 Sep	S11 May	S11 Jun	S11 Jul	S11 Aug	S11 Sep
Amiota albilabris	0	1	5	21	5	0	0	1	0	1
Amiota alboguttata	0	1	1	0	3	0	0	0	0	0
Amiota rufescens	0	0	12	8	0	0	0	1	1	0
Amiota subtusradiata	0	0	0	0	0	0	0	0	0	0
Gitona distigma	2	10	2	1	8	0	0	2	0	0
Leucophenga maculata	0	0	0	0	6	0	0	0	0	1
Leucophenga quinquemaculata	20	5	0	4	2	13	1	0	0	8
Phortica semivirgo	1	0	0	1	0	1	1	0	0	3
Chymomyza amoena	0	0	0	0	0	0	0	0	0	1
Chymomyza caudatula	0	0	0	0	0	0	0	0	0	0
Chymomyza costata	4	0	0	0	0	1	2	0	2	0
Chymomyza fuscimana	0	0	0	0	0	0	0	0	0	1
Drosophila busckii	0	0	0	0	0	0	0	0	0	0
Drosophila funebris	0	0	0	0	0	0	0	0	0	0
Drosophila histrio	12	0	0	2	2	8	5	0	4	33
Drosophila hydei	0	0	0	0	0	0	0	0	0	0
Drosophila immigrans	0	0	0	0	1	0	0	0	0	2
Drosophila kuntzei	0	0	0	0	0	1	0	0	0	7
Drosophila littoralis	0	0	0	0	0	0	0	0	0	0
Drosophila melanogaster	0	0	0	0	1	0	0	0	0	0
Drosophila obscura	17	25	28	134	78	22	25	28	85	132
Drosophila phalerata	9	1	0	3	3	15	2	0	4	33
Drosophila subobscura	2	14	15	68	14	0	8	14	27	15
Drosophila testacea	187	4	2	12	27	221	16	5	33	307
Drosophila transversa	99	25	1	50	3	19	7	2	37	14
Drosophila tristis	1	0	0	0	1	0	0	0	0	0
Hirtodrosophila confusa	0	0	0	0	0	0	0	0	1	1
Hirtodrosophila toyohiokadai	0	0	0	0	0	1	0	0	0	0
Scaptodrosophila rufifrons	1	1	3	3	6	0	1	2	5	10
Scaptomyza pallida	0	0	0	0	3	0	0	0	0	1
**Total individuals**	**355**	**87**	**69**	**307**	**163**	**302**	**68**	**55**	**199**	**570**

Table S4. Complete results of restricted permutation analyses and sensitivity analyses of drosophilid community composition.

Effect / comparison	Data representation	pseudo-F	df effect	df residual	partial R²	P	Permutation scheme
Season	Square-root counts	5.8057	4	40	0.3673	0.0002	4,999 restricted + observed
Season	Relative abundance	8.1305	4	40	0.4484	0.0002	4,999 restricted + observed
Season	Raw counts	4.0370	4	40	0.2876	0.0002	4,999 restricted + observed
burned vs control	Square-root counts	11.5967	1	49	0.1914	0.0182	55 exact allocations
three groups	Square-root counts	6.8957	2	48	0.2232	0.0130	4620 exact allocations
once vs twice	Square-root counts	1.8176	1	39	0.0445	0.3333	84 exact allocations
burned vs control	Relative abundance	10.2255	1	49	0.1727	0.0182	55 exact allocations
three groups	Relative abundance	5.8986	2	48	0.1973	0.0286	4620 exact allocations
once vs twice	Relative abundance	1.4077	1	39	0.0348	0.5119	84 exact allocations
burned vs control	Raw counts	10.3883	1	49	0.1749	0.0182	55 exact allocations
three groups	Raw counts	6.3732	2	48	0.2098	0.0069	4620 exact allocations
once vs twice	Raw counts	2.0733	1	39	0.0505	0.2857	84 exact allocations

Additional repeated-measures tests. Species richness differed among months (Friedman $\chi^2 = 18.373$, $P = 0.00104$), whereas total drosophilid abundance did not show a significant common monthly pattern across sites (Friedman $\chi^2 = 6.886$, $P = 0.142$).

Notes. Square-root-transformed counts constitute the primary community analysis. Relative-abundance analyses remove differences in total trap catch and test compositional differences alone. Raw-count analyses are retained as a sensitivity analysis. For between-site tests, all monthly observations from a site retained the same permuted group label, so repeated observations were never treated as independent spatial replicates. Exact P values were calculated from all allowable whole-site label allocations. For the seasonal test, month labels were permuted only within sampling sites (4,999 restricted permutations).
